# Initial assembly steps of a translocase for folded proteins

**DOI:** 10.1038/ncomms8234

**Published:** 2015-06-11

**Authors:** Anne-Sophie Blümmel, Laura A. Haag, Ekaterina Eimer, Matthias Müller, Julia Fröbel

**Affiliations:** 1 Institute of Biochemistry and Molecular Biology, ZBMZ, University of Freiburg, 79104 Freiburg, Germany; 2 Spemann Graduate School of Biology and Medicine (SGBM), University of Freiburg, 79104 Freiburg, Germany; 3 Faculty of Biology, University of Freiburg, 79104 Freiburg, Germany

## Abstract

The so-called Tat (twin-arginine translocation) system transports completely folded proteins across cellular membranes of archaea, prokaryotes and plant chloroplasts. Tat-directed proteins are distinguished by a conserved twin-arginine (RR-) motif in their signal sequences. Many Tat systems are based on the membrane proteins TatA, TatB and TatC, of which TatB and TatC are known to cooperate in binding RR-signal peptides and to form higher-order oligomeric structures. We have now elucidated the fine architecture of TatBC oligomers assembled to form closed intramembrane substrate-binding cavities. The identification of distinct homonymous and heteronymous contacts between TatB and TatC suggest that TatB monomers coalesce into dome-like TatB structures that are surrounded by outer rings of TatC monomers. We also show that these TatBC complexes are approached by TatA protomers through their N-termini, which thereby establish contacts with TatB and membrane-inserted RR-precursors.

## Abstract (web summary)

The twin-arginine translocation complex consists of TatA, TatB and TatC subunits and transports folded proteins across cellular membranes. Here, using photocrosslinking, the authors show that TatB monomers form dome-like structures that are surrounded by TatC monomers enabling lateral access of TatA.

## Graphical abstract

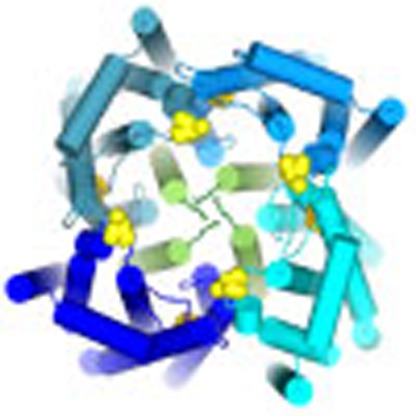

Tat (twin-arginine translocation) machineries exist in the plasma membranes of bacteria and archaea and the thylakoid membranes of plant chloroplasts, across which they transport folded proteins that contain the consensus motif S-R-R-x-F-L-K in their signal sequences^1^,^2^,^3^,^4^,^5^. These Tat machineries are constructed from TatA- and TatC-type membrane proteins. TatA is an N-out single-span membrane protein with a closely spaced amphipathic helix. A functionally distinct paralogue, termed TatB, is expressed in Gram-negative bacteria and plants. *Enterobacteria* express even a third paralogue, TatE, which can functionally replace TatA. TatC is a hexahelical protein, whose helices are kinked and tilted forming a concave wall structure^6^,^7^.

TatB and TatC function as substrate receptor complexes, while TatA is required to achieve translocation, a process that requires a transmembrane H^+^-gradient as sole energy source. It has been suggested that multiple TatA monomers associate either via their transmembrane^8^ or their amphipathic helices^9^,^10^ to form size-fitting pores for the Tat substrates. This idea has, however, been challenged by studies questioning the necessary redundancy of TatA^11^,^12^,^13^. Alternatively, TatA was proposed to mediate translocation by destabilizing the lipid bilayer^14^,^15^.

In live *E. coli* cells, Tat machineries were recently demonstrated to assemble on demand^16^,^17^ but the details how the TatABC subunits associate to form an active translocase remain poorly understood. Using an extensive site-specific photocrosslinking approach, we have now shed light on the likely architecture of the TatBC–substrate complex and how the assembly of TatA might be initiated. Our results suggest that dome-like TatB structures form the core of intramembrane substrate-binding cavities that are surrounded by outer rings of TatC monomers. We propose that TatA laterally enters those substrate–TatBC complexes.

## Results

### RR-precursors contact TatC deep inside the membrane

**Figure 1:**
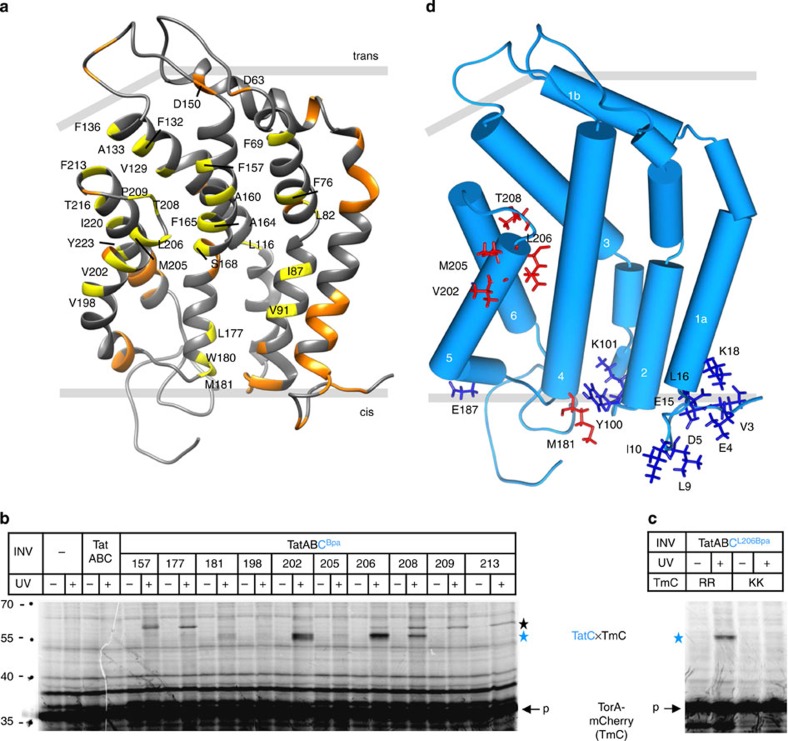
Contacts between TatC and a membrane-inserted RR-precursor. (**a**) Model of *E. coli* TatC based on the structure of *A. aeolicus* TatC^7^ using PDB code 4B4A. Indicated in yellow are all amino acids replaced by Bpa in this study, and in orange those of a previous analysis^19^. (**b**) The model RR-precursor TorA-mCherry was synthesized and radioactively labelled by *in vitro* transcription/translation in the absence or presence of inverted *E. coli* inner membrane vesicles (INV). In addition to TatA and TatB, INV contained either wild-type TatC (TatABC) or the indicated Bpa variants of TatC (TatABC^Bpa^). In samples labelled (+), crosslinking was initiated by irradiation with ultraviolet light. Radiolabelled translation products were separated by SDS–PAGE and visualized by phosphorimaging. Indicated are the positions of molecular size standard proteins (left-hand side), the TorA-mCherry (TmC) precursor (p), and the crosslinked TatC–TmC complex (blue star). The black star marks a ultraviolet light-dependent crosslink of unknown nature between TorA-mCherry and several TatC variants. (**c**) comparing crosslinking of the L206Bpa variant of TatC to wild-type TorA-mCherry (RR) and to a mutant with an inactive signal peptide (KK). (**d**) Highlighted in red are all residues that in **b** showed distinct contacts to TorA-mCherry when replaced by Bpa, and in blue the precursor contact sites identified in our previous study^19^. The helices of TatC are numbered.

We recently demonstrated that in the absence of TatB, TatC is sufficient to insert RR-precursor proteins into the plasma membrane of *E. coli* even to a point where they become prematurely processed by signal peptidase^18^. We now sought to follow the track of such an inserted RR-precursor on the TatC molecule. To this end, we incorporated the photo-activatable crosslinker *p*-benzoyl-phenylalanine (Bpa) into 28 novel positions of *E. coli* TatC (Fig. 1a; shown in yellow) that had not been analysed previously^19^ (Fig. 1a; orange residues). Inside-out inner-membrane vesicles (INV) were prepared from *E. coli* strains expressing the individual Bpa-containing variants of TatC and were incubated with the *in vitro* synthesized and radioactively labelled Tat model substrate TorA-mCherry^18^. Results obtained with 10 of the TatC variants are shown in Fig. 1b. Ultraviolet light-dependent, radiolabelled crosslinking products of the size of 1:1 complexes between TorA-mCherry and TatC (55 kDa, blue star) appeared when Bpa had been incorporated at the positions 181, 202, 205, 206 and 208 of TatC. The specificity of these TatC–TorA-mCherry contacts is indicated by the failure to obtain them when using a transport-incompetent KK-mutant of TorA-mCherry (Fig. 1c). Several residues gave rise to ultraviolet light-dependent crosslinks that were ∼10 kDa larger (Fig. 1b; black star). They could not clearly be distinguished from an unspecific band and therefore remain of unknown origin at this point.

The location of the five residues of TatC yielding the 1:1 complexes with TorA-mCherry is illustrated in Fig. 1d (red side chains). Position 181 in the transmembrane helix (TM) 4 of TatC is located on the *cis*-side of TatC, much like the previously identified^19^,^20^ recognition site for RR-signal peptides (Fig. 1d; blue side chains). The other residues crosslinking to TorA-mCherry cluster towards the opposite membrane side of TatC, that is, on the distal part of TM5 (202, 205, 206) and the intervening loop between TM5 and 6 (208) (Fig. 1d; red side chains). Due to the supposed membrane-thinning effect of the short TM5 and TM6 of TatC^6^,^7^, the location of these contact sites invoke a largely transmembrane insertion of RR-precursors exactly as it was previously deduced from their accessibility to signal peptidase. Despite the depth of insertion, this step proceeds independently of the H^+^-motive force (Supplementary Fig. 1).

### Each TatC monomer contains two contact sites for TatB

**Figure 2:**
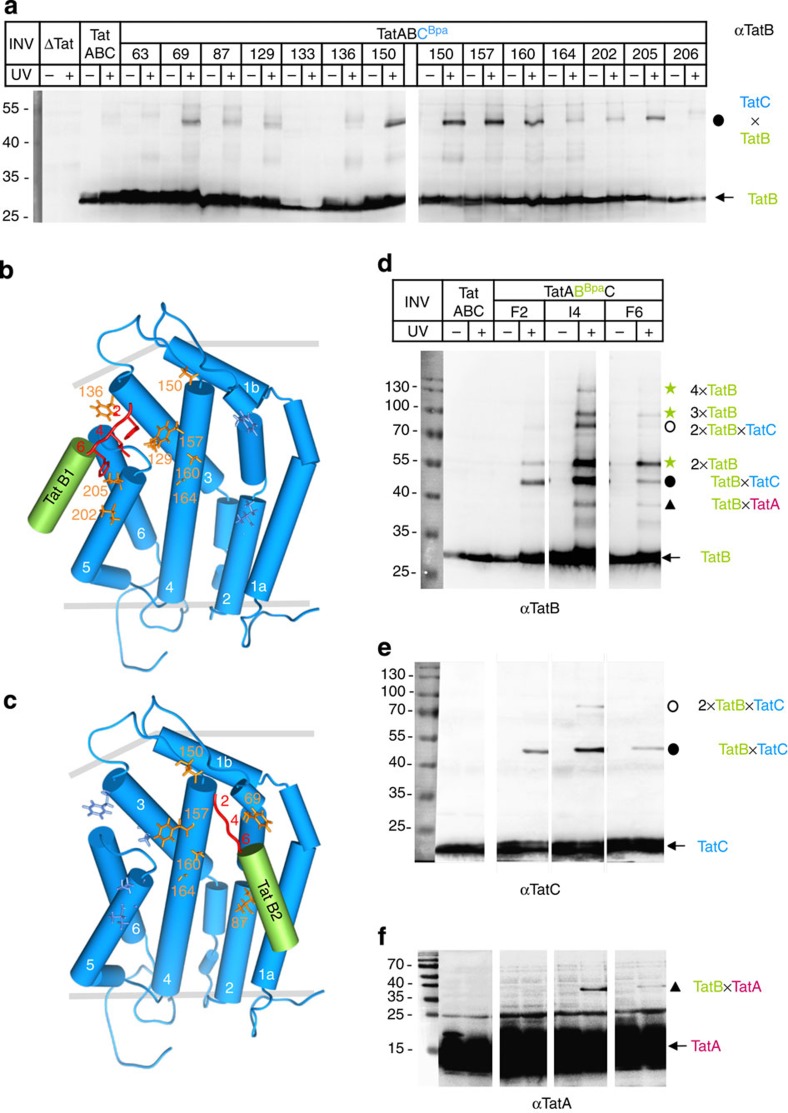
Identification of mutual intramembrane contacts between TatB and TatC. (**a**) Membrane vesicles (INV) containing no Tat proteins (ΔTat), wild-type TatABC and TatAB together with the indicated Bpa variants of TatC (TatABC^Bpa^) were irradiated with ultraviolet light (+) or mock treated (−) and then probed for TatB-cross-reactive adducts to TatC. Shown are western blots developed with anti-TatB antibodies (αTatB). Included are all of the TatCBpa variants shown in Fig. 1a in yellow, which yielded 1:1 complexes between TatB and TatC (black dot). (**b**,**c**) Models of *E. coli* TatC highlighting in orange residues that crosslinked to TatB according to **a**. The TatB-reactive sites cluster on two areas on the front of TatC invoking binding of two TatB monomers (TatB1, TatB2). The transmembrane helices (green) and the N-termini (red) of the two TatB monomers were modelled onto the TatC structure. (**d**) INV carrying the indicated Bpa variants in the N terminus of TatB were probed for TatB adducts on western blots using antibodies against TatB (αTatB). Indicated are predicted N-terminally colligated oligomers of TatB (green stars), as well as complexes between TatB and TatC (black dots and circles) and TatA (triangles). (**e**) As in **d**, using antibodies against TatC (αTatC). (**f**) As in **d**, using antibodies against TatA (αTatA).

We next probed the membrane vesicles harbouring Bpa variants of TatC also for contacts between TatB and TatC. Crosslinking was induced by irradiating the INV with ultraviolet light, and the obtained adducts were analysed for the presence of TatB on western blots (Fig. 2a). Ultraviolet light-induced and TatB-cross-reactive adducts of ∼50 kDa, indicative of 1:1 TatBC complexes, appeared for residues located in the TM5 of TatC (202 and 205, black dot), consistent with the previously proposed alignment of the TM5 of TatC with the TM of TatB^7^,^21^. More prominent crosslinks of this size were, however, obtained for the Bpa157 variant and, as already previously recognized^19^, for Bpa150. As illustrated in Fig. 2b, these positions would not be in direct reach of the transmembrane helix of TatB lining up with the TM5 of TatC, but rather close to the more distally located amino (N) terminus of TatB, which according to recent NMR studies^22^ is depicted as a non-helical structure in Fig. 2b (in red).

Other positions of TatC yielding 1:1 adducts with TatB (Fig. 2a; black dot) cluster towards the distal end of TM3 of TatC (129 and weakly 136) and the central part of TM4 (160 and 164), where they would still be in some proximity to the N terminus of a TatB molecule bound to TM5 (Fig. 2b). On the contrary, the TatB-reactive residues 69 and 87 (TM2) cluster in the right-hand half of TatC (Fig. 2c). These sites would be too remote to explain crosslinking to the same TatB molecule and therefore invoke a second TatB monomer binding to the same TatC molecule via TM2 and potentially also TM4 (TatB2 in Fig. 2c).

### TatB and TatA N-termini mediate inter-subunit contacts

To verify these contacts between the *trans*-sided area of TatC and the N terminus of TatB, we incorporated Bpa into the three N-terminal positions of TatB indicated in Fig. 2b,c (red numbers). As shown by western blotting using an anti-TatB antiserum (Fig. 2d), INV carrying these Bpa variants yielded a number of adducts to the 30 kDa TatB when irradiated with ultraviolet light. Two of the crosslinks (black dot and circle, 50 and 80 kDa in size) were recognized also by anti-TatC antibodies (compare Fig. 2d with Fig. 2e) thus representing the expected TatB–TatC adducts. The 50 kDa adduct (black dot) numerically corresponds to a 1:1 TatBC complex (30 kDa+23 kDa), whereas the mass of the 80 kDa adduct (circle) suggests a complex of one TatC (23 kDa) and 2 TatB (2 × 30 kDa) molecules. The formation of such ternary complexes is in total agreement with the two concomitant TatB-binding sites identified on a single TatC molecule. In addition, three TatB adducts were obtained (Fig. 2d; green stars; most prominent for the I4 variant of TatB) that were recognized neither by antibodies directed against TatC (Fig. 2e) nor TatA (Fig. 2f). Because of the documented self-association tendency of TatB^23^,^24^ and the lack of any information that functional Tat translocases involve quantitative amounts of non-Tat proteins, we assume that those TatB-cross-reactive adducts represent homo-oligomers of TatB, that by approximate mass could be dimers, trimers and tetramers. These findings would be consistent with the N terminus of TatB being involved in connecting TatB and TatC within TatBC complexes.

In addition to being in the proximity of TatC, we found the N terminus of TatB also crosslinked to TatA (adduct of ∼38 kDa in Fig. 2d,f, triangles). Likewise, when Bpa was incorporated into TatA at equivalent N-terminal positions as in TatB, it formed crosslinks to TatC as well as oligomers of TatA (Supplementary Fig. 2). The combined crosslinking results therefore provide strong evidence for a coalescence of the three TatABC subunits in the *trans*-sided half of the bilayer with the N-termini of TatA and TatB possibly being involved in the association of the three Tat subunits.

### RR-signal peptides contact oligomers of TatB and TatA

**Figure 3:**
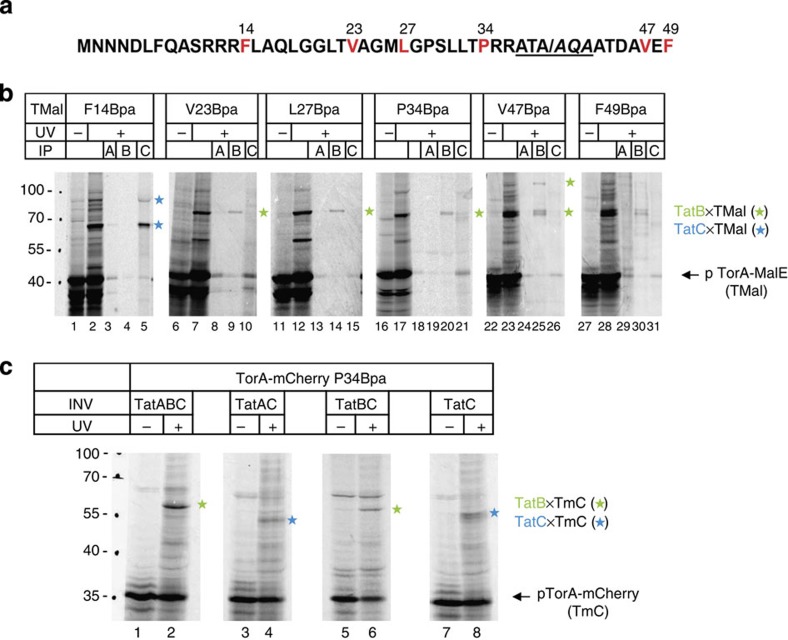
A TatBC-walled insertion site for RR-precursors. (**a**) N-terminal sequence shared by the model RR-precursors TorA-MalE and TorA-mCherry with the residues highlighted that were replaced by Bpa. The six amino acids flanking the cleavage site (slash) of the TorA signal peptide are underlined. (**b**) The indicated Bpa variants of TorA-MalE (TMal) were synthesized *in vitro* in the presence of membrane vesicles containing the TatABC proteins and crosslinking was initiated by ultraviolet light irradiation (for details see legend to Fig. 1b). One aliquot of each irradiated reaction (+) was directly analysed by SDS–PAGE and phosphorimaging (lanes 2, 7, 12, 17, 23, 28), while others were first immuno-precipitated (IP) using antibodies against TatA, TatB, TatC (lanes A,B,C). Crosslinking products recognized by anti-TatB and anti-TatC antibodies are indicated by the green and blue stars, respectively. No TatA-immuno-reactive adducts were obtained. (**c**) The P34Bpa variant of TorA-mCherry (TmC) was synthesized *in vitro* in the presence of membrane vesicles (INV) containing the indicated Tat proteins. Crosslinking and labelling of the adducts are as in **b**.

As noted above, the TM5 of TatC seems to be a common docking site for RR-precursors and TatB raising the question of a direct contact between TatB and RR-precursors in this *trans*-sided area of TatC. If so, TatB might shield the cleavage site of a deeply inserted RR-precursor and thereby prevent premature exposure to signal peptidase as recently suggested^18^. To experimentally verify such a juxtaposition of RR-precursor and TatB, we incorporated Bpa into the TorA signal sequence at several positions reaching from the RR-consensus motif until beyond the cleavage site (Fig. 3a). The Bpa variants of the model Tat substrate TorA-MalE^19^ were synthesized *in vitro* in the presence of INV containing all three Tat subunits and crosslinking was initiated by irradiation with ultraviolet light (Fig. 3b). When inserted at position F14, Bpa crosslinked TorA-MalE (TMal) to TatC as verified by immuno-precipitation of the crosslinking products (Fig. 3b; lanes 2 and 5, blue stars). This confirms the previously established recognition of the RR-containing consensus motif by TatC^20^,^25^,^26^. In addition to the ∼60 kDa TatC-TMal adduct, an ∼90 kDa ultraviolet light-dependent crosslinking product was recognized by the anti-TatC antibodies (Fig. 3b; lanes 2 and 5, upper blue star) suggesting that two closely spaced precursors^27^ contact one TatC molecule. In contrast, all the sites selected further downstream in the TorA signal sequence (V23, L27, P34) and beyond the signal sequence cleavage site (V47 and F49) crosslinked to TatB (Fig. 3b; green stars). For the V47Bpa variant of TMal, an additional larger TatB-cross-reactive adduct was obtained (lane 25) that could reflect binding of two precursors to the same TatB monomer either individually or as a linked dimer. If the Bpa variants of the TorA signal sequence were incubated with INV that selectively lacked TatB, the originally TatB-reactive sites were now found in proximity to TatC. This is demonstrated in Fig. 3c for the P34Bpa variant of TorA-mCherry (TmC), which yielded a prominent adduct of the size of a TatB–TmC complex whenever TatB was present (lanes 2 and 6, green stars). In its absence, however, the smaller adduct to TatC was obtained (lanes 4 and 8, blue stars). These results are consistent with a TatBC-walled insertion cavity, in which the TorA signal sequence would be closer to TatB so that interactions with the more external TatC become detectable only in the absence of TatB.

**Figure 4:**
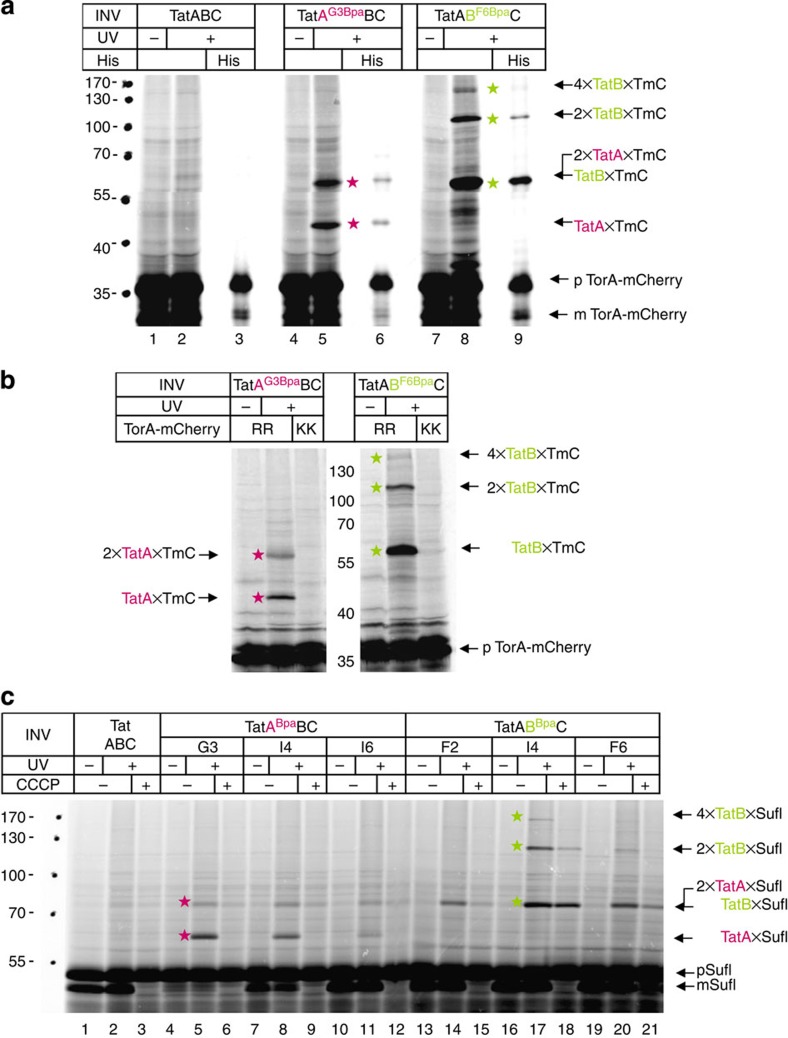
Membrane-inserted RR-precursors touch at the N-termini of oligomeric TatB, subsequently also of TatA. (**a**) *In vitro* synthesis of the model RR-precursor TorA-mCherry in the presence of membrane vesicles (INV) containing wild-type TatABC, TatBC plus the G3Bpa variant of TatA and TatAC plus the F6Bpa variant of TatB. Crosslinking between these N-terminal Bpa variants and TorA-mCherry was initiated by ultraviolet light irradiation (+). Adducts of TorA-mCherry (TmC) to TatA (red stars) and TatB (green stars) could be purified via the His-tag of TorA-mCherry (His). The positions of the precursor (p) and the signal sequence-less form (m) of TorA-mCherry are indicated. (**b**) As in **a**, comparing crosslinking of the N-termini of TatA and TatB to wild-type TorA-mCherry (RR) and to its transport-incompetent mutant (KK). (**c**) As in **a**, using the three indicated N-terminal Bpa variants of TatA and TatB and the natural *E. coli* Tat substrate pSufI. In addition, the effects of dissipating the H^+^-motive force by the uncoupler CCCP on the crosslinking behaviour of pSufI to the N-termini of TatA and TatB are shown (red and green stars). The effectivity of CCCP is demonstrated by the failure of TatABC-INV (and others) to process the precursor of SufI (pSufI) to the signal sequence-less mature form (mSufI; lane 3).

Next, we incubated INV that contained N-terminal Bpa variants of TatA or TatB with *in vitro* synthesized and radioactively labelled Tat substrates. In full agreement with a deep insertion, ultraviolet light-dependent adducts of TorA-mCherry to both N-termini of TatA and TatB (Fig. 4a; red and green stars) were observed. Notably, multiple adducts were obtained in either case. Because all of them were radioactively labelled and could be purified through the His-tag attached to TorA-mCherry (lanes 6 and 9), they all must contain the RR-precursor. Those multiple contacts are functionally relevant, because they were all abrogated when a transport-incompetent KK-mutant of TorA-mCherry was used (Fig. 4b). Very similar results were obtained for the natural *E. coli* Tat substrate pSufI (pFtsP) and three N-terminal Bpa variants of TatA and TatB each (Fig. 4c). These results clearly indicate that after insertion into the Tat translocase, RR-precursor molecules can associate simultaneously with the N-termini of multiple TatB and TatA molecules.

The N-termini of TatB thus do not only interconnect TatB monomers (Fig. 2d) but also contact an RR-signal peptide as part of an oligomeric TatB structure (Fig. 4). Given the slanted orientation of TatB's TM (Fig. 2b) the combined data therefore seem to suggest a dome-like structure made from the N-termini and TMs of several TatB monomers that thereby would encapsulate the RR-signal sequences of Tat substrate proteins.

The contacts depicted in Fig. 4 that RR-precursors formed with the N-termini of TatB and TatA must, however, reflect two different stages of interaction because they were discernible by their dependence on the H^+^-motive force. Dissipation of the H^+^-motive force by the uncoupler cyanide *m*-chlorophenyl-hydrazone (CCCP) blocks the Tat-dependent transport of the precursor of SufI (pSufI) thereby preventing cleavage of pSufI to mature SufI (Fig. 4c; lanes 2 and 3, mSufI). Although CCCP caused the TatA-adducts of SufI to disappear virtually completely (lanes 6, 9, 12, red stars), it only attenuated crosslinking to TatB, with the adducts containing multimers of TatB being apparently more affected than those to a single TatB molecule (lanes 15, 18, 21, green stars). Since the initial binding of RR-precursors to the Tat translocase proceeds independently of the H^+^-motive force, it is likely that the interaction of a single precursor molecule with the N terminus of TatB precedes its contact with that of TatA, which obviously requires the H^+^-motive force. Similarly, the recruitment of more than one N terminus of TatB to the signal sequence seems, at least in part, to be dependent on the H^+^-motive force. Though requiring the H^+^-motive force, the N-terminal association of a TatA dimer with an RR-precursor must occur before translocation, because it was observed although the incorporation of Bpa into position G3 of TatA largely abolished translocation (Fig. 4c; missing mSufI band in lanes 4–6; see also Supplementary Fig. 3). These findings suggest that in the presence of the H^+^-motive force, an RR-precursor after being inserted into the TatBC binding cavity can be approached by protomers of TatA from within the membrane and thereby establish contacts to the N terminus of TatA, its TM and amphipathic helix^28^.

### RR-peptides are sequestered within a TatBC-walled cavity

**Figure 5:**
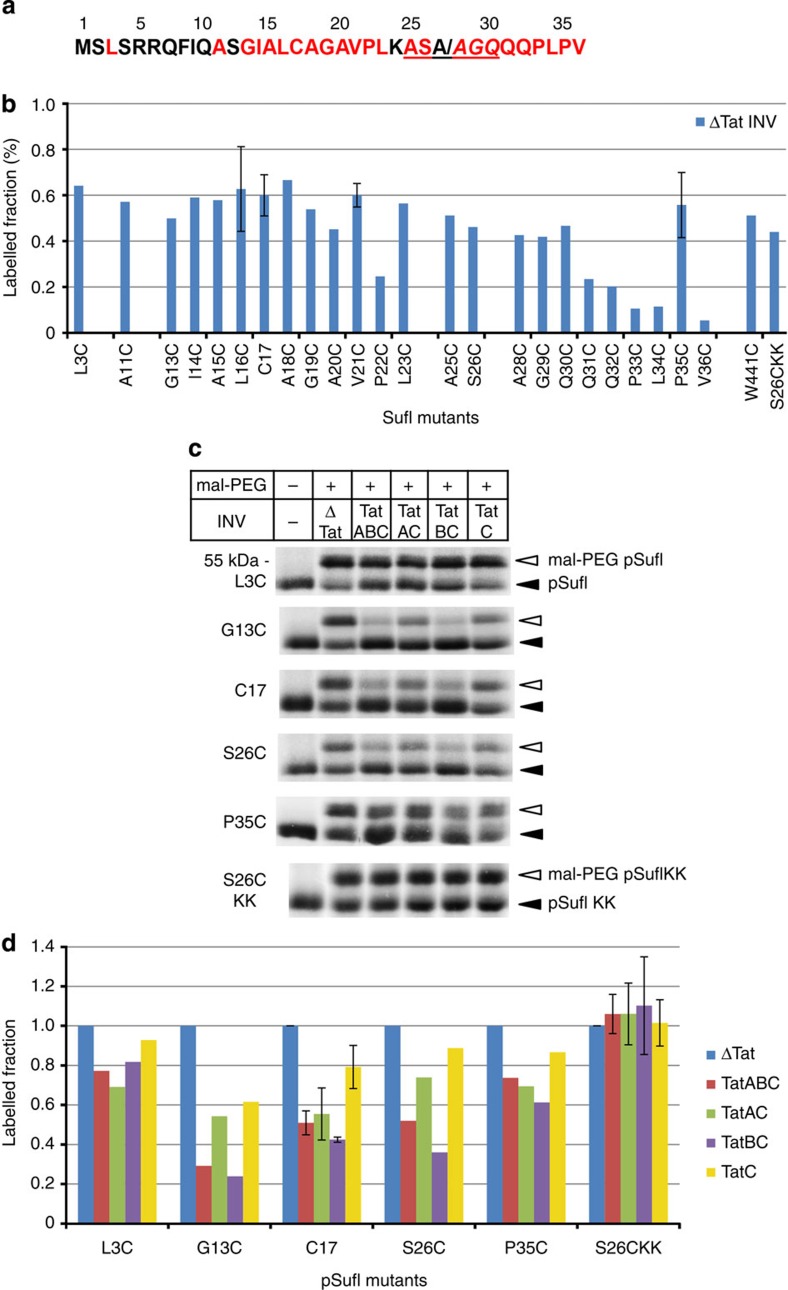
RR-signal peptides are sequestered within a TatBC-made insertion cavity. (**a**) N-terminal sequence of the natural RR-precursor pSufI highlighting the residues that were exchanged against Cys. The six amino acids flanking the cleavage site (slash) of the signal peptide are underlined. (**b**) Relative labelling by mal-PEG of the indicated Cys mutants of pSufI following their *in vitro* synthesis and incubation with ΔTat-INV, that is, under conditions, in which they could not interact with the Tat translocase. Indicated are the mean ratios between labelled and unlabelled pSufI species obtained from two or three parallel experiments shown in **c** (lane ΔTat). Where an error bar is shown, the data represent the mean ± standard deviation of three parallel experiments. Otherwise, the mean of two experiments is shown. Supporting data from each individual experiment are shown in Supplementary Table 1. About 50–60% of each Cys mutant became thus labelled with mal-PEG, except for P22 (for unknown reasons) and residues in the early mature region of SufI (due to folding). (**c**) The Cys variants of pSufI and one transport-incompetent KK-derivative, which are specified on the left-hand side, were synthesized and radiolabelled *in vitro*, incubated with INV containing the indicated Tat proteins, and reacted with mal-PEG. Shown are the PEGylated (white arrowhead) and unmodified (black arrowhead) species of pSufI following SDS–PAGE and phosphorimaging. (**d**) Quantitative data obtained from two or three parallel experiments shown in **c**. Values were normalized to the labelling efficiency obtained in the presence of ΔTat-INV. Where an error bar is shown, the data represent the mean ± standard deviation of three parallel experiments. Otherwise, the mean of two experiments is shown. Supporting data from each individual experiment are shown in Supplementary Table 2. Protection against PEGylation was obtained for positions within the signal sequence of pSufI but not for flanking residues. Protection by INV was more pronounced in the presence of TatB than in its absence.

To provide independent experimental evidence for the sequestration of an RR-signal peptide within an intramembrane insertion cavity, we probed for its accessibility towards a branched form of maleinimido-polyethylene glycol (mal-PEG). To this end, single Cys mutants of pSufI (Fig. 5a) were synthesized *in vitro*, subsequently incubated with INV containing various combinations of the TatABC subunits, labelled with mal-PEG and analysed by SDS–polyacrylamide gel electrophoresis (SDS–PAGE) and phosphorimaging. The experimental conditions were adjusted so as to largely prevent membrane permeation of mal-PEG and removal of pSufI from TatABC due to transport in, and cleavage by, the membrane vesicles. In this way, 50–60% of the molecules of most Cys-bearing pSufI mutants became labelled with mal-PEG, when they were incubated with INV that lacked the TatABC proteins (Fig. 5b; Supplementary Table 1). Labelling of pSufI-C22 was markedly impaired for unknown reasons, whereas the reduced accessibility of most residues beyond position 30 was due to their sequestration within the folded part of pSufI, as verified by an increase in PEGylation of residue V36 in the presence of 8 M urea and the unrestricted labelling of the surface-exposed^29^ residue W441 (Supplementary Fig. 4).

If the Cys variants of pSufI were, however, incubated with vesicles containing the TatABC proteins, PEGylation dropped significantly for mutants that carried a Cys between positions 11 and 26. This is exemplarily illustrated for residues 13, 17 and 26 in Fig. 5c,d (TatABC; Fig. 5d displays quantitative data obtained from two to three parallel experiments each; a full data set is provided in Suppplementary Table 2). A complete protection by TatABC-INV against PEGylation was not to be expected, since not all pSufI molecules interacted with our INV. In contrast to residues 13, 17, 26, Cys-labelling in areas flanking the core of the signal sequence (C3 and C35) turned out to be much less affected by the presence of TatABC-containing INV. The combined findings would be consistent with a hairpin-like insertion of the SufI signal sequence into a cavity of the Tat translocase, which restricts the access of mal-PEG to residues within the inserted region. In support of this conclusion, protection against PEGylation was not observed for the KK variant of preSufI C26 under any condition (Fig. 5c,d; S26CKK). Reduced accessibility of residues 13–26 of the SufI signal sequence to mal-PEG was also observed for membrane vesicles containing only TatB and TatC (Fig. 5c,d; TatBC) confirming that the primary insertion site of an RR-precursor is provided by TatB and TatC only. When TatB was missing (Fig. 5c,d; TatAC and TatC), protection against labelling with mal-PEG was diminished yet still occurred, demonstrating again the membrane insertase activity of TatC. Formation of an enclosed cavity that most extensively restricts access to mal-PEG, however, requires the cooperation of TatC with TatB.

### TatB influences TatC–TatC and TatC–TatA interactions

**Figure 6:**
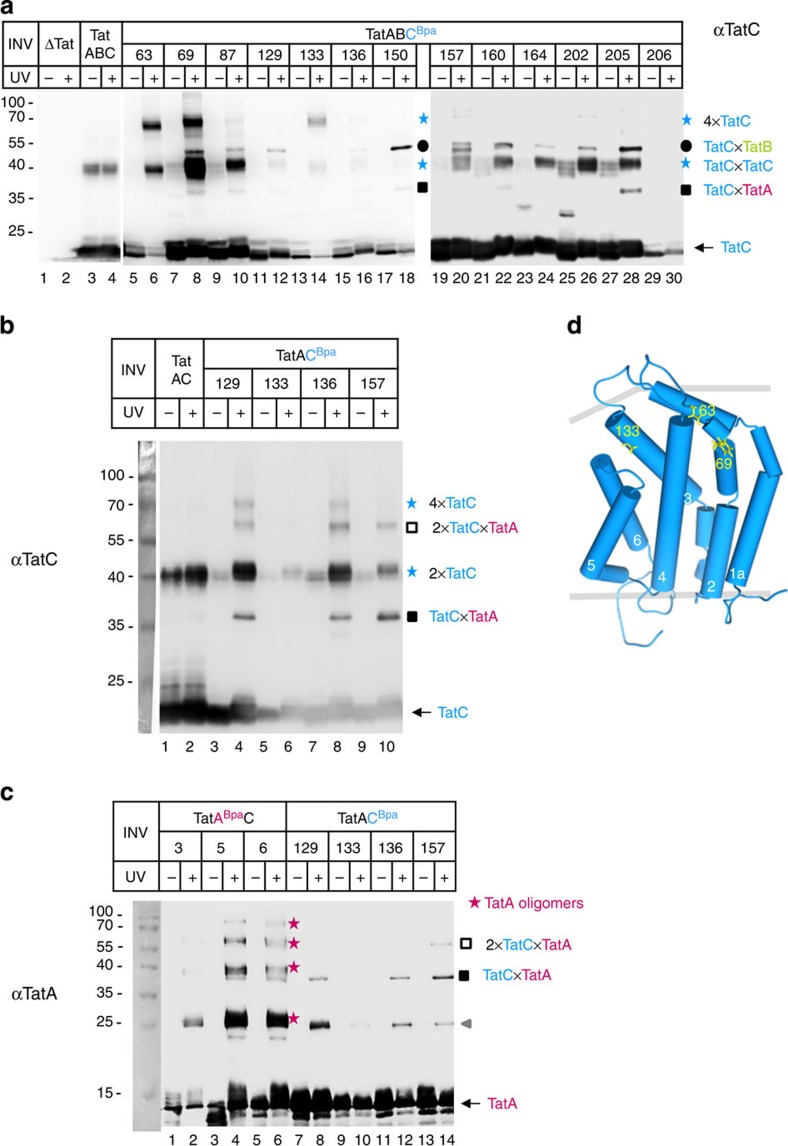
Homo-oligomerization of TatC and its contacts to TatA are influenced by TatB. (**a**) Membrane vesicles containing the indicated Bpa variants of TatC (Fig. 2a) were probed for inter-TatC crosslinks (blue stars). Shown are western blots decorated with anti-TatC antibodies (αTatC). TatB–TatC adducts (black dots) had been identified as such in Fig. 2a and TatA–TatC adducts (squares) are addressed in **b** and **c**. (**b**,**c**) As in **a**, except that membrane vesicles used were devoid of TatB. Ultraviolet light-dependent adducts to the indicated Bpa variants of TatC were probed by antibodies against TatC (αTatC) and TatA (αTatA) revealing the 37 kDa TatA–TatC complexes (black squares). The same adduct was also obtained when Bpa was incorporated into the N terminus of TatA (**c**, lanes 4 and 6). TatA oligomers (red stars) crosslinked through the N terminus of TatA were also obtained. In addition, TatA forms dimers independently of Bpa (**c**, lanes 8–14, grey arrow head). Open squares mark an adduct that by size consists of two TatC and one TatA molecule. (**d**) Model of the *E. coli* TatC structure highlighting in yellow the three residues that yielded pronounced tetramers of TatC (**a**).

When the Bpa variants of TatC, which had been analysed for crosslinks to TatB (Fig. 2a), were screened for the occurrence of inter-TatC contacts, the results depicted in Fig. 6a were obtained. Besides the 50 kDa adducts (black dots), which due to their cross-reactivity with anti-TatB antibodies (Fig. 2a) represent 1:1 TatB–TatC complexes, additional 40 and 70 kDa adducts were obtained (blue stars) that were only recognized by the anti-TatC antibodies. The 40 kDa, most likely dimeric form of TatC was even observed for Bpa-free TatC when INV contained high amounts of TatC (Fig. 6a, lanes 3 and 4, blue star), similar to what has been observed previously^30^,^31^. Otherwise, most of the Bpa variants of TatC shown in Fig. 6a dimerized upon activation by ultraviolet light (lanes 5–10 and 21–30, lower blue stars). A considerable amount of TatC63Bpa, however, prevailed as a 70 kDa adduct (lane 6, upper blue star), that is, most likely as a tetramer^19^. The same adduct was obtained for the 69Bpa and 133Bpa variants of TatC (lanes 8 and 14), indicating that distinct residues on the *trans*-side of TatC (Fig. 6d) seem to play an important role in mediating its oligomerization.

As shown in Fig. 6a, some of the Bpa variants of TatC also yielded a rather faint adduct of ∼37 kDa (black squares). For the 150Bpa variant (lane 18) we had previously identified this adduct as a 1:1 TatA–TatC complex^19^. Assuming that interactions between TatC and TatA might be hampered by the presence of TatB, we constructed several of the Bpa-bearing TatC variants also on a plasmid that selectively lacked the *tatB* gene. As shown in Fig. 6b for the TatC variants Bpa129, 136 and 157, more pronounced TatC adducts of ∼37 kDa (black square) were now obtained, which were recognized by anti-TatA antibodies (Fig. 6c, black squares). In further support of this adduct being a TatA–TatC complex, a crosslinking product of identical electrophoretic mobility was obtained when Bpa had been incorporated into N-terminal residues of TatA rather than into TatC (Fig. 6c, lanes 4 and 6, black square). The residues of TatC that in the absence of TatB yielded prominent TatAC crosslinks (additional ones are shown in Supplementary Fig. 5b,c), are either part of the TatB-binding sites depicted in Fig. 2b,c or are located in their vicinity. The combined data of Fig. 6 and Supplementary Fig. 5 therefore indicate an overlap between the binding sites for TatA and TatB on TatC, suggesting that TatB might control the access of TatA to TatC.

Conspicuously, the TatC133Bpa variant lost its ability to form TatC tetramers in the absence of TatB (compare Fig. 6a, lane 14 to Fig. 6b, lane 6; see also Supplementary Fig. 5a; lane 8 and Fig. 5b; lane 6). Thus, TatB also seems to be an important determinant for the complexation of TatC.

### Identification of contact sites between TatC protomers

**Figure 7:**
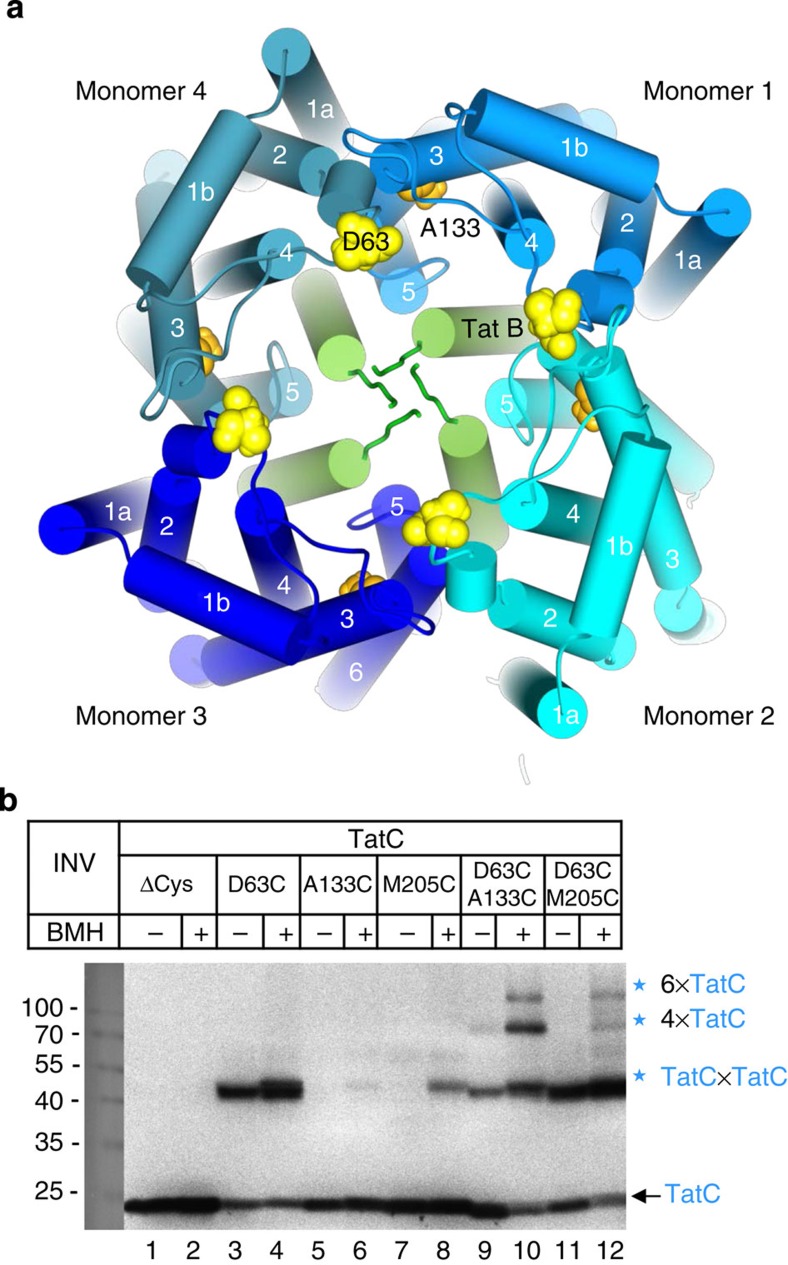
Formation of circular TatBC oligomers through specific contact sites of TatC. (**a**) Model of a TatBC tetramer looking down the membrane normal from the *trans*-side of the Tat translocase. Residues D63 and A133 of each TatC monomer are highlighted. (**b**) Membrane vesicles (INV) containing TatAB and either a Cys-less mutant of TatC (TatCΔCys) or the indicated single and double Cys mutants of TatC were either incubated with the sulfhydryl specific homobifunctional crosslinker BMH or mock treated. Proteins were dissolved in DTT-free SDS–PAGE loading buffer and analysed for the formation of TatC oligomers (blue stars) by SDS–PAGE and western blotting using anti-TatC antibodies.

On the basis of the two binding sites for TatB identified on the front side of a single TatC molecule (Fig. 2b,c) and the contacts of TatB monomers through their N-termini (Fig. 2d,e), it seems likely that the TatBC binding cavity for RR-precursors consists of a dome-like shell of TatB monomers that is surrounded by an outer shell made from TatC protomers. In Fig. 7a, we have modelled such a structure using four TatB and TatC molecules each and displayed it in top view looking down from the *trans*-side of the membrane. Each TatC monomer is approached by two TatB molecules, the TMs of which lie parallel to TM5 and cross TM2 and TM4 of TatC, respectively. In this model, the residues D63 and A133 of TatC (highlighted in yellow and orange), which crosslinking revealed as hotspots for tetramerization (Fig. 6a), would be positioned in some proximity. To verify this model, we replaced both residues either individually or jointly by Cys in an otherwise Cys-free variant of TatC^24^. We also constructed an M205C variant, because Bpa at this position of TatC caused much less tetramerization than D63 or A133 (Fig. 6a). INV containing those Cys variants of TatC were either incubated with the bifunctional Cys crosslinker Bismaleimidohexane (BMH) or mock treated and then probed by immuno-blotting against TatC antibodies for the formation of TatC complexes. Depending on the position, the single Cys mutants formed disulfides (Fig. 7b; lanes 1–8) reinforcing the dimerization tendency of TatC. If, however, INV contained the double Cys mutant D63C/A133C, larger adducts appeared that by size correspond to tetrameric and hexameric TatC complexes (lanes 9, 10). The formation of these oligomers was strongly favoured by BMH but was not obtained through direct oxidation by sodium tetrathionate (Supplementary Fig. 6), which is in line with a certain distance between D63 and A133 as proposed by the model in Fig. 7a. Because the tetramers and hexamers could not be obtained with the single Cys mutants, they must arise from mixed disulfides between D63 and A133. Two TatC molecules bridged through a single D63–A133 crosslink could then use their free D63C and A133C residues each to crosslink to a second dimer as illustrated in Fig. 7a. Inclusion of a third TatC dimer could then lead to a circular hexamer and so forth. Furthermore, the model places residue M205 at the tip of TM5 of TatC in a position that would be consistent with the weak oligomerization observed for the D63C/M205C double Cys mutant (Fig. 7b; lane 12).

## Discussion

Our extended crosslinking analysis of TatC has now revealed two different contact areas for TatB on the concave face of each TatC monomer. One of them clusters around the TM5 of TatC. In accordance with previous studies^7^,^20^,^21^, binding of TatB to this site likely results in the peripheral apposition of TatB's TM to the TM5 of TatC. In addition, we demonstrate here that central positions in the TM4 and TM2 of TatC also establish contacts with TatB. Their distance to the TM5-based binding area invokes the attachment of a second TatB molecule on the concave face of each TatC (Fig. 2b,c). A likely explanation for the provenance of this second TatB monomer was that it stemmed from a neighbouring TatBC protomer, to which it would be fastened via the TM5 of TatC. Hence in the TatBC receptor complex, TatBC protomers would be linked via the TMs of TatB, each one intercalating between TM2/4 and TM5 of two adjacent TatC molecules (Fig. 7a). This model is strongly supported by our Bpa crosslinking analysis of TatC, identifying the distal end of TM3 (TatC133) and the loop between TM1 and TM2 (TatC63, TatC69) as hotspots involved in TatC oligomerization and revealing the requirement of TatB for the oligomerization of TatC through residues such as TatC133. In direct proof of the assumption that these residues of TatC mediate contact between two adjacent TatC monomers in the TatBC receptor complex, we demonstrate by Cys–Cys crosslinking between residues TatC133 and TatC63 the formation of dimeric, tetrameric and hexameric TatC assemblies. Adjacent TatBC protomers might also be linked together via the N-termini of TatB, because we detected up to tetrameric TatB structures concatenated through the N-terminal six amino acids of TatB. Simply for this reason, we selected a tetrameric structure for our model in Fig. 7a but our results would also be fully compatible with dimeric^32^,^33^, heptameric^34^ and octameric TatBC^35^ assemblies. In summary, TatBC protomers would oligomerize through both, homonymous (N-termini of TatB, area around TatC133-TatC63) and heteronymous (multiple TatBC contacts) linkages.

Our results are indicative of the substrate-binding site of the Tat translocase being a closed cavity that deeply extends into the membrane. As illustrated in the cross-section of our TatBC model (Fig. 7a), a core structure consisting mostly of TatB TMs would be surrounded by an outer ring of TatC monomers. In contrast to previous models^24^,^36^, the inner TatB layer is now predicted to form a dome-like structure, due to crosslinking of the individual TatB monomers through their N-termini and in agreement with the slanted position of each TatB TM suggested by its alignment with the similarly tilted TM5 of TatC^7^. Numerous data indicate that this dome-like inner shell of TatB monomers establishes the immediate contact with the Tat substrate, thereby forming a deeply membrane-embedded signal sequence-binding cavity with a closed roof that limits exit to the *trans*-side of the membrane. Thus, we found an RR-signal sequence crosslinked to TatB almost along its entire length, including the cleavage site and the early mature region of a Tat substrate, which is in line with previous reports^25^,^26^. In addition, we show here that RR-precursors simultaneously associate with the N-termini of up to four TatB molecules and we previously detected contacts between the TMs of up to three TatBs and a single RR-precursor molecule^36^. Moreover, we recently demonstrated that TatB prevents an RR-precursor, which was inserted only by TatC, to become exposed to the *trans*-sided signal peptidase and to thereby prematurely lose its signal peptide^18^. The inner TatB layer does, however, not form an entirely closed wall around inserted RR-precursors. This is because we found a section of TatC encompassing *trans*-sided residues of TM5 and the TM5/6 loop to crosslink to an inserted RR-precursor even in the presence of TatB. In full agreement, crosslinks between an RR-precursor and residue Val270 in the distal end of TM5 of chloroplast TatC were described, which made the authors conclude that substrate insertion must occur on the concave faces of adjacent TatC monomers^32^. A protrusion of TM5 of TatC into the inner TatB ring suggesting direct contact with substrate, actually becomes apparent in the model of Fig. 7a. On the contrary, the external location of TatC in the TatBC binding cavity is consistent with several crosslinks between an inserted RR-signal sequence and TatC that became manifest only after removing TatB (Fig. 3c). A deep insertion of RR-precursors into a binding cavity jointly formed by TatB and TatC is further consistent with the previous isolation of distinct suppressor mutations in *trans*-sided domains of TatB and TatC that alleviate the translocation defects of mutated RR-consensus motifs^37^,^38^,^39^.

Because *cis*-domains of TatC constitute the recognition site for the RR-consensus motif of Tat substrates^19^,^20^, RR-signal peptides remain N-terminally anchored to the surface of the Tat translocase and are threaded in a loop-like (inverted) configuration into the TatBC binding cavity. Consistent with such a loop formation, we picked up interactions between the early mature region of an RR-precursor and TatC (Fig. 3b; lane 31) as well as between the surface-oriented residue Glu187 of TatC (Fig. 1a) and RR-precursors^19^. Likewise, chloroplast TatC was demonstrated to crosslink to an RR-signal peptide distal to its H-domain^32^. By demonstrating differential accessibility of RR-precursors to the membrane-impermeable alkylating reagent mal-PEG, we have provided independent experimental evidence for an RR-signal peptide forming a hairpin within the TatBC binding cavity. Although protection of an RR-signal peptide against labelling with mal-PEG could in principle result from a mere lipid insertion, in combination with our crosslinking data, it rather supports sequestration within a TatBC binding cavity. The mal-PEG approach further revealed that inserted RR-signal peptides are largely inaccessible from the *cis*-side of the Tat translocase. This was not necessarily expected given the water-filled funnel reaching into the *cis*-part of TatC, which has been deduced from MD simulations of TatC in a lipid bilayer^6^. We propose that the closure of the cavity is initially achieved to a large extent by the amphipathic helices of the TatB monomers that according to NMR measurements seem to be oriented in a rather fixed angle with regard to the TMs^22^. This could result in a straddling position of the amphipathic TatB helices across the *cis*-opening of the signal sequence-binding cavity. Such an orientation of TatB would fit with crosslinks of TatB to the substrate recognition site of TatC^19^ and to folded substrate domains^36^. Because supposedly each TatBC protomer binds one Tat substrate^35^, RR-precursors once inserted into an oligomeric TatBC binding cavity must be accommodated in close proximity to each other as demonstrated previously^27^,^32^, which would explain the crosslinking that we observed between two RR-precursors and one TatC or one TatB monomer (Fig. 3b; lanes 5 and 25).

The N-termini of TatB and TatA have now been identified as pivotal players in the assembly of an active Tat translocase. According to our crosslinking data, the N terminus of TatB does not only protrude far into the *trans*-sided half of TatC, where it might concatenate TatB monomers, but also forms contacts with TatA. On the other hand, we found also TatA to homo-oligomerize via its N-terminal amino acids, which is in accordance with previous data^40^. Thus the N-termini of TatA and TatB might fulfil important functions in the mutual association of the subunits of the Tat translocase.

In agreement with the results of other experimental approaches^41^,^42^, past^19^,^28^,^32^ and the present crosslinking analyses established direct contacts between the N terminus and TM of TatA and distinct *trans*-sided parts of TatC. The contacts between TatC and TatA increased in number and intensity when TatB was missing. Moreover, the TatA binding sites on TatC largely overlapped with those for TatB. These findings suggest that TatB might control the access of TatA to TatC. Furthermore, our crosslinking analysis revealed that two TatA molecules contact a single RR-precursor molecule via their N-termini, which would be consistent with a TatA oligomer associating with a TatABC-bound RR-precursor. Since these contacts were strongly enhanced in the presence of the H^+^-motive force, it is likely that they follow conformational rearrangements of the Tat subunits^43^. In fact, Ken Cline's group recently demonstrated the translocation of the thylakoidal TatA (Tha4) molecule from a TatB-like peripheral position near the TM5 to a cleft between TM2 and TM4 on the concave face of TatC, which required substrate binding and the H^+^-motive force^32^. The interpretation of those findings by the authors—in combination with our model of the TatBC–substrate insertion complex—predicts that TatA monomers peripherally insert into the TatBC cupola in between adjacent TatC monomers, thereby extending its diameter and creating space for an internal oligomerization of TatA.

## Methods

### Plasmids

Plasmids used in this study are listed in Supplementary Table 3. Plasmids p8737 and p8737-tatAC were used to construct amber stop codon mutations of *tatA, tatB* and *tatC* by mutagenizing PCR^36^ using the pairs of forward and reverse primers listed in Supplementary Table 4. Plasmids pPJ3, pPJ5 and pPJ11 were used to introduce amber stop codon mutations into the genes encoding TorA-mCherry, KK-TorA-mCherry and TorA-MalE, respectively by mutagenizing PCR^44^ using the pairs of forward and reverse primers listed in Supplementary Table 4. To construct plasmid pEJ, the *sufI* coding sequence was amplified by PCR using plasmid pKSMSufI-RR as template and the oligonucleotide primers SufI NdeI for and SufI XhoI rev (Supplementary Table 4). The PCR fragment and the recipient vector pET22b+ were digested with NdeI and XhoI and ligated following dephosphorylation of the vector. To generate plasmid pETRick encoding the KK variant of SufI, plasmid pEJ was site-specifically mutagenized according to the QuikChange site-directed mutagenesis kit protocol (Stratagene) using the primers SufI KK F and SufI KK R (Supplementary Table 4). Plasmids pLJ1 and pLJ2 were constructed by using pEJ and pETRick as templates, respectively. The cysteines at position 17 and 295 of SufI were mutated to alanines by mutagenizing PCR using the primers SufI C17A and SufI C295A (Supplementary Table 4). Plasmids pLJ1, pLJ2 and pPJ3 were used to introduce the Cys codons TGT and TGC into the genes encoding SufI and TorA-mCherry according to the QuikChange site-directed mutagenesis kit protocol (Stratagene) using the primers listed in Supplementary Table 4. In the same way, Cys variants of TatC were constructed using plasmid pUNITATCC4 as template DNA and the primers listed in Supplementary Table 4.

### *In vitro* reactions

The RR-precursor proteins TorA-mCherry, TorA-MalE and pSufI were synthesized by *in vitro* transcription/translation using the plasmids indicated in Supplementary Table 3. Cell extracts were prepared^45^ from *E. coli* strain SL119 (ref. 46) or Top10 (Invitrogen) transformed with plasmid pSup-BpaRS-6TRN(D286R) to express amber stop codon mutants of RR-precursors^36^. These cell extracts were used to synthesize radioactively labelled proteins from plasmid DNA by coupled transcription/translation in 50 μl reactions^45^. INV were added 10–15 min after starting the synthesis reaction and incubated for 20 min at 37 °C. To dissipate the H^+^-motive force of the vesicles, 0.1 mM CCCP was added together with INV. Protein translocation into INV was assayed via proteinase K protection^28^. Crosslinking was initiated by irradiating samples with ultraviolet light for 20 min on ice^28^. For purification of the *in vitro* synthesized RR-precursor proteins containing a carboxy (C)-terminal His-tag and their Tat adducts, proteins were precipitated with TCA, dissolved in DTT-free SDS–PAGE loading buffer, treated with 8 M urea and applied to Ni-Sepharose^47^.

For immuno-precipitation^25^, samples were denatured in 1% SDS for 10 min at 95 °C when immuno-precipitating TatA and TatB, and for 10 min at 45 °C when immuno-precipitating TatC. After centrifugation, supernatants were incubated for 3 h at 4 °C with Protein A-Sepharose previously loaded with antisera for 90 min at 4 °C. After the final washing step, antigen–antibody complexes were released by shaking in SDS–PAGE loading buffer^45^ for 10 min at 37 °C and 1,400 r.p.m.

For Cys-labelling with mal-PEG (Maleinimidyl-PEG(4)-[PEG(10)-OMe]3; IRIS Biotech) 75 μl aliquots of coupled transcription/translation reactions were incubated with 0.8 mM puromycin (final concentration) for 10 min at 37 °C. After ultracentrifugation for 15 min at 208,000*g*, 50 μl of each supernatant were diluted 5-fold with 50 mM potassium phosphate buffer pH 7.5, and 27.1 μl of this solution were incubated with 1 μl INV for 5 min at 5 °C. Afterwards, 2 μl of 11.29 mM mal-PEG diluted with H_2_O from a 100 mM stock solution in DMSO were added to yield a final concentration of 0.75 mM and incubated for 10 min at 5 °C. Reactions were stopped with 10% TCA.

For Cys crosslinking, 5 μl INV were diluted with 45 μl INV buffer^45^. One half of this solution was mock treated with 0.25 μl DMSO and the other incubated for 1 h at 37 °C with 0.25 μl 20 mM Bismaleimidohexane (Thermo Fischer) prepared in DMSO. Proteins were precipitated with 10% TCA and re-dissolved in DTT-free SDS–PAGE loading buffer.

Proteins were resolved on SDS–PAGE using 8, 10 or 15% polyacrylamide gels^45^.

### Membrane vesicles

INV were prepared^45^ from *E. coli* strains BL21(DE3)* (Novagen) or BL21(DE3)ΔTat (B. Ize and T. Palmer, personal communication) transformed with plasmid p8737 (TatABC-INV), p8737-tatAC (TatAC-INV), pFAT75CHΔA (TatBC-INV) or pFAT588 (TatC-INV), respectively. For Cys-labelling experiments, ΔTat-INV were prepared from strain DADE (MC4100, Δ*tatABCDE*). TatABC- and TatAC-INV containing Bpa variants of TatA, TatB and TatC were prepared from *E. coli* strains BL21(DE3)* or BL21(DE3)ΔTat transformed with plasmid pEVOL-pBpF and derivatives of p8737 and p8737-tatAC that carried the individual *tatA, tatB* or *tatC* amber stop codon mutations. Expression of *tat* genes was induced with 1 mM IPTG. To suppress amber stop codons, expression from pEVOL-pBpF was induced with 0.1% arabinose. Bpa (0.25 mM) was added together with IPTG.

To detect contacts between the Tat subunits, 5 μl of each INV preparation (∼100 A_280_ units per ml) were diluted with 95 μl INV Puffer^45^, irradiated with ultraviolet light for 20 min on ice, TCA-precipitated and resuspended in 100 μl SDS–PAGE loading buffer. Five microlitres were used for western blotting against TatA and 20 μl each when probed with TatB and TatC antisera.

### Graphics

Molecular graphics and analysis were performed with the UCSF Chimera package (http://www.cgl.ucsf.edu/chimera).

### Image processing

The original, uncropped images of all SDS–polyacrylamide gels and western blots are shown in Supplementary Fig. 7.

## Additional information

**How to cite this article:** Blümmel, A.-S. *et al*. Initial assembly steps of a translocase for folded proteins. *Nat. Commun.* 6:7234 doi: 10.1038/ncomms8234 (2015).

## Supplementary Material

Supplementary Information Supplementary Figures 1-7, Supplementary Tables 1-4 and Supplementary References
