# Supplementary material for: Initial assembly steps of a translocase for folded proteins
Source: Nat Commun. 2015 Jun 11;6:7234. doi: 10.1038/ncomms8234 (PMC4490388; doi:10.1038/ncomms8234)
Supplement: Supplementary Information — Supplementary Figures 1-7, Supplementary Tables 1-4 and Supplementary References [file ncomms8234-s1.pdf]

SUPPLEMENTARY INFORMATION

Supplementary Figures

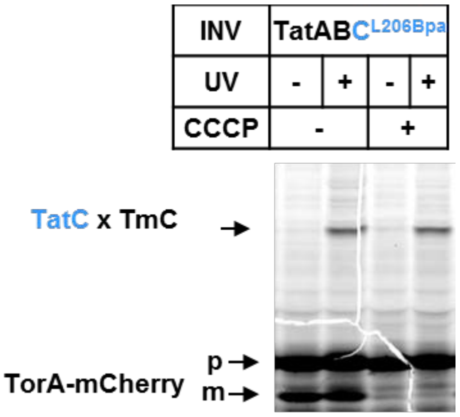

Supplementary Figure 1. Contacts between TatC and a membrane-inserted RR-precursor do not require the H^{+}-motive force. The model RR-precursor TorA-mCherry was synthesized and radioactively labeled by *in vitro* transcription/translation in the presence of *E. coli* inner membrane vesicles containing TatA, TatB, and the L206Bpa variant of TatC. In samples labeled (+), cross-linking was initiated by irradiation with UV light. Radiolabeled translation products were separated by SDS-PAGE and visualized by phosphorimaging. Cross-linking between TatC and TorA-mCherry (TatC x TmC) was not influenced by the uncoupler CCCP, which, however, blocks transport into the vesicles, as indicated by a lack of conversion of the precursor (p) to the mature form (m) of TorA-mCherry.

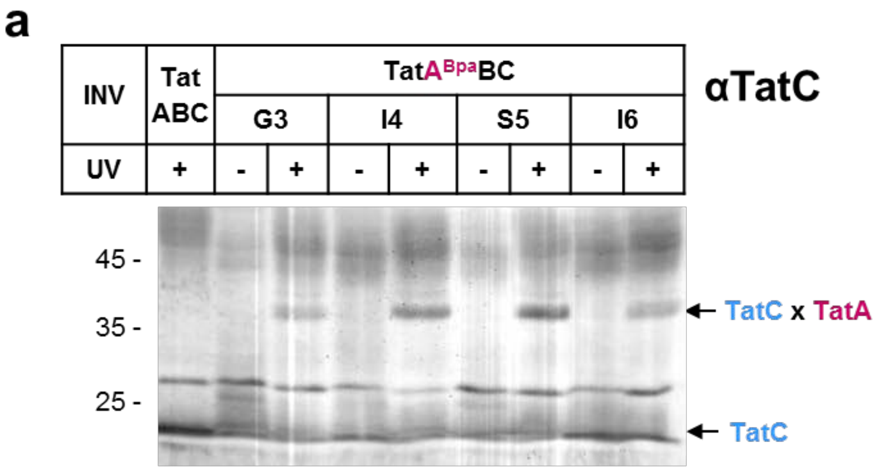

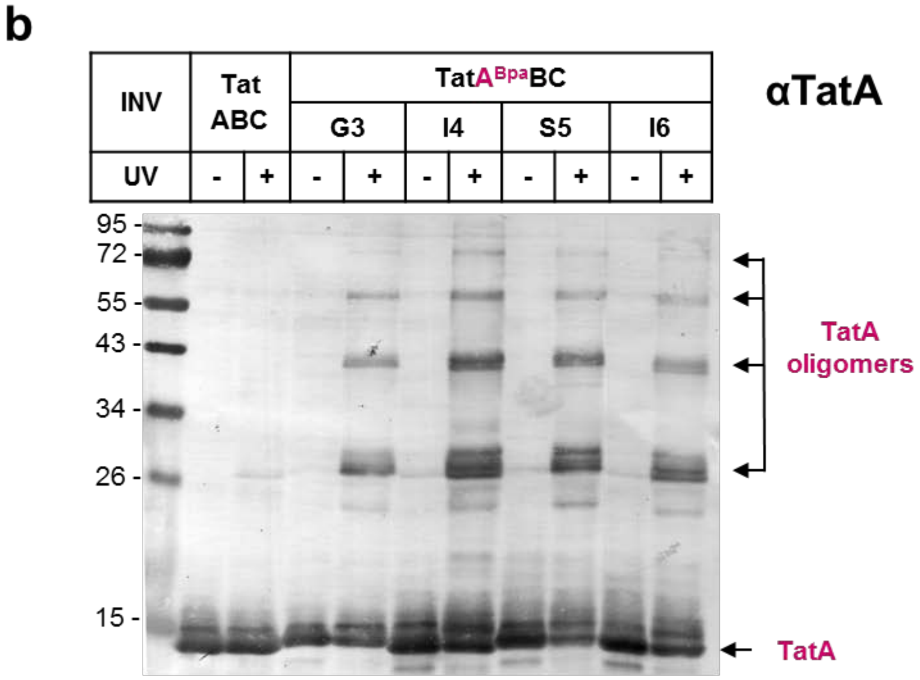

Supplementary Figure 2. The N-terminus of TatA cross-links to TatC and mediates oligomerization of TatA. (**a, b**) Inner membrane vesicles (INV) carrying the indicated Bpa variants in the N-terminus of TatA were probed for TatA adducts on Western blots using antibodies against TatC (αTatC) and TatA (αTatA). Cross-linking was induced by irradiation with UV light (+). Indicated are complexes between TatA and TatC as well as N-terminally colligated oligomers of TatA. TatABC, Bpa-free vesicles.

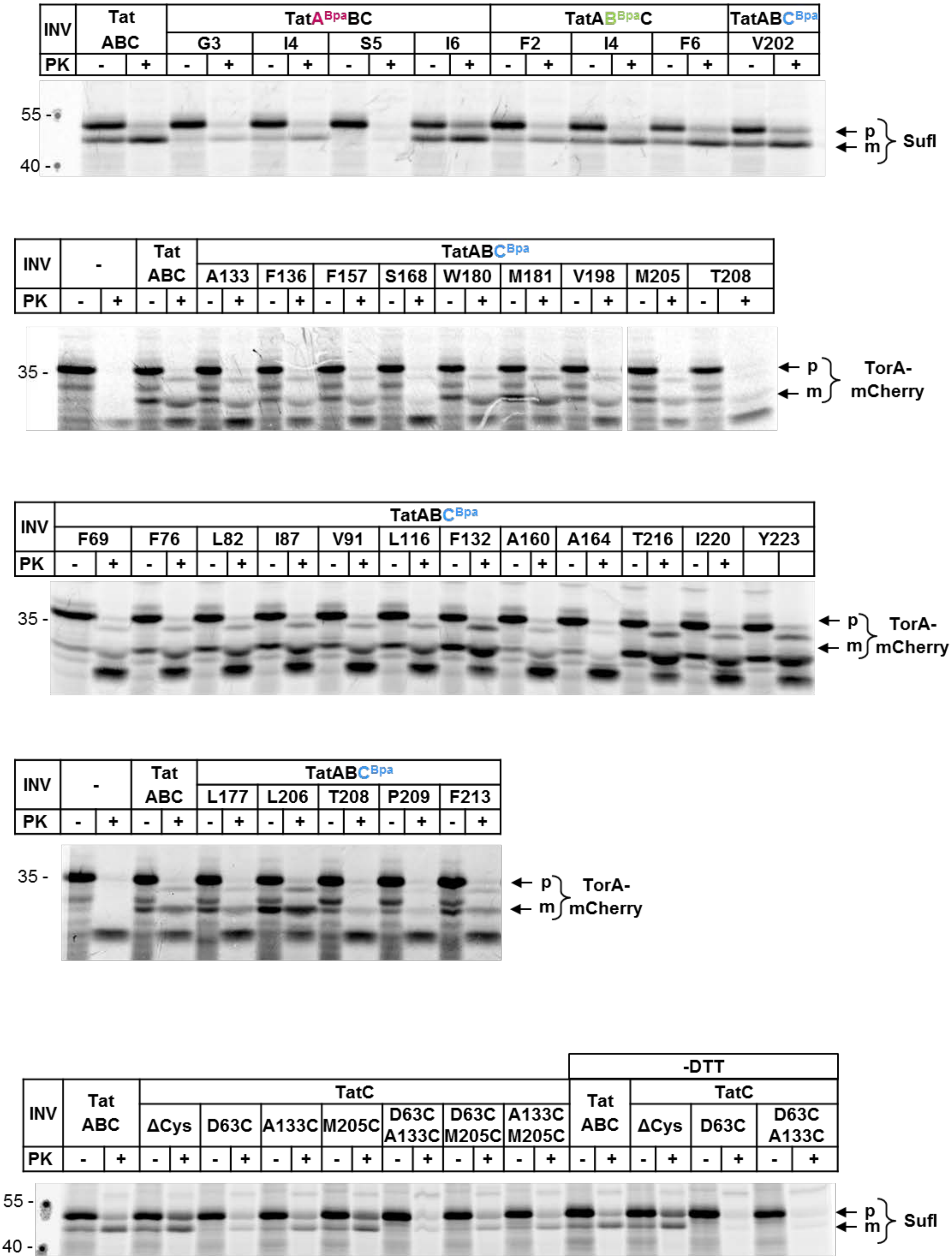
INV
Tat ABC
TatA^Bpa BC
G3 I4 S5 I6
TatAB^Bpa C
F2 I4 F6
TatABC^Bpa
V202
PK - + - + - + - + - + - + - + - + - +
55 -
40 -
p
m
SufI
INV
-
Tat ABC
TatABC^Bpa
A133 F136 F157 S168 W180 M181 V198 M205 T208
PK - + - + - + - + - + - + - + - + - + - + - +
35 -
p
m
TorA-mCherry
INV
TatABC^Bpa
F69 F76 L82 I87 V91 L116 F132 A160 A164 T216 I220 Y223
PK - + - + - + - + - + - + - + - + - + - + - +
35 -
p
m
TorA-mCherry
INV
-
Tat ABC
TatABC^Bpa
L177 L206 T208 P209 F213
PK - + - + - + - + - + - + - +
35 -
p
m
TorA-mCherry
-DTT
INV
Tat ABC
TatC
ΔCys D63C A133C M205C D63C A133C D63C M205C A133C M205C
Tat ABC
TatC
ΔCys D63C D63C A133C
PK - + - + - + - + - + - + - + - + - + - + - + - +
55 -
40 -
p
m
SufI

Supplementary Figure 3. Translocation activity of membrane vesicles carrying Bpa-containing Tat variants used in this study. The RR-precursors TorA-mCherry and pSufI were synthesized *in vitro* in the presence of membrane vesicles (INV) containing the wild-type TatABC proteins or any of the indicated Bpa variants of TatA (A^{Bpa}), TatB (B^{Bpa}), or TatC (C^{Bpa}). Shown are the radioactively labeled translation products obtained without (-) or after (+) digestion with proteinase K (PK). The positions of the precursors (p) and mature forms (m) of TorA-mCherry and SufI are indicated. (Bottom panel), vesicles were prepared from *E. coli* strain BL21(DE3)ΔTat expressing extra-chromosomally a Cys-less mutant of TatC (TatCΔCys) carrying single or double cysteine mutations where indicated. Assays performed in the total absence of DTT (- DTT) revealed that TatC dimers, which formed through disulfide bridges at position D63, are inactive.

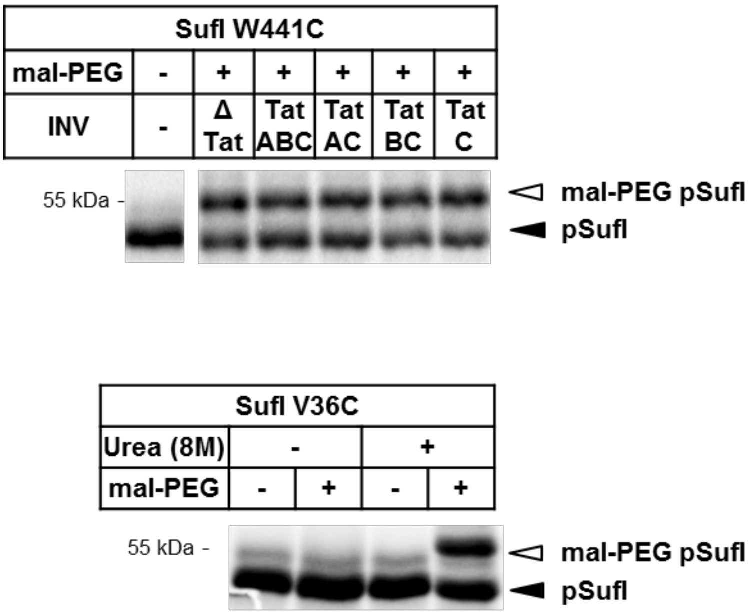

Supplementary Figure 4. Control experiments to Figure 5. The surface exposed residue W441 of SufI is accessible to mal-PEG, whereas residue V36 in the early mature region can be labeled only after denaturation of SufI with 8M urea. Experimental details are as described in the legend to Fig. 5 and in the Materials section.

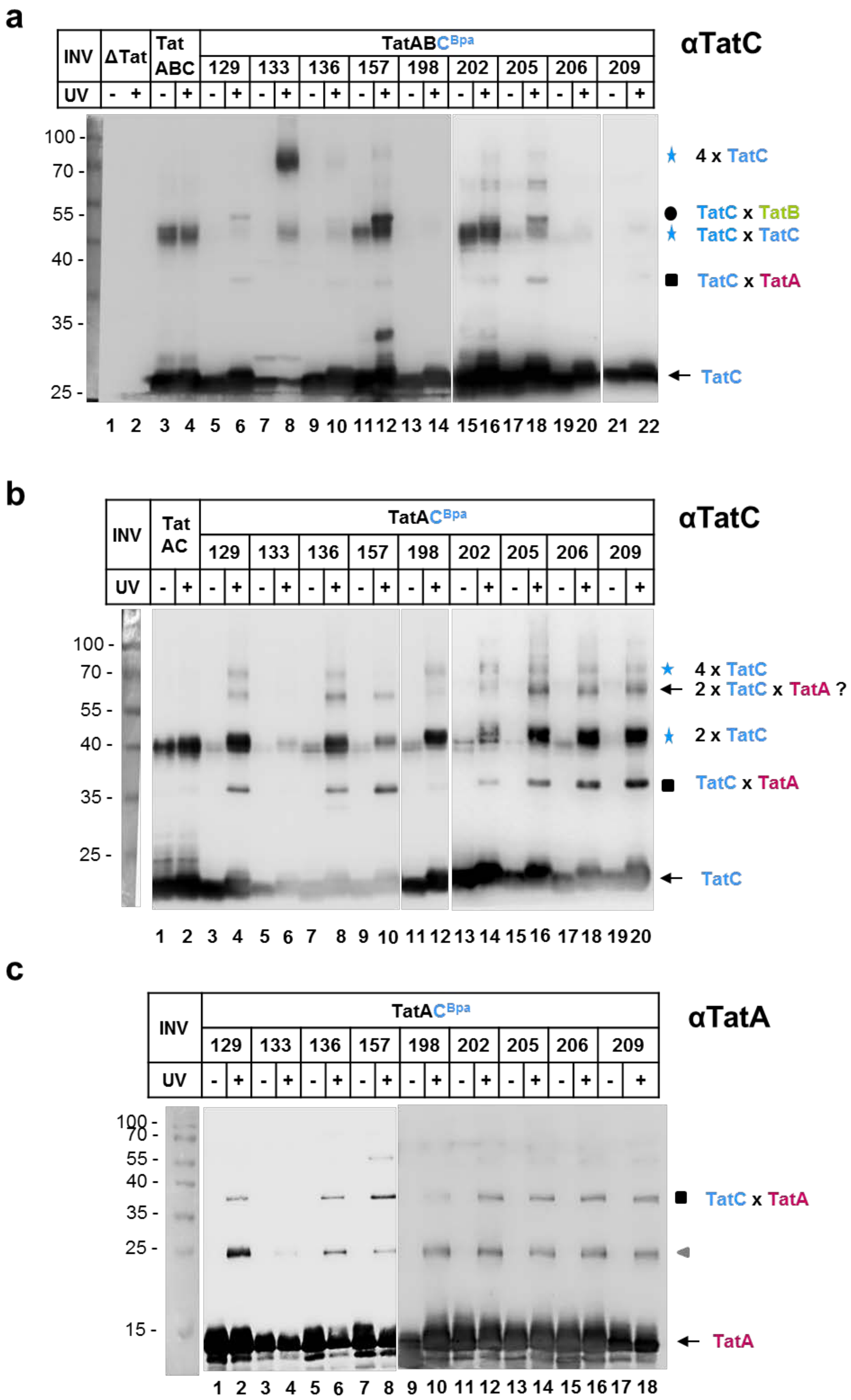
a
INV
ΔTat
Tat ABC
TatABC^{Bpa}
129
133
136
157
198
202
205
206
209
UV
αTatC
100 -
70 -
55 -
40 -
35 -
25 -
4 x TatC
TatC x TatB
TatC x TatC
TatC x TatA
TatC
1 2 3 4 5 6 7 8 9 10 11 12 13 14 15 16 17 18 19 20 21 22
b
INV
Tat AC
TatAC^{Bpa}
129
133
136
157
198
202
205
206
209
UV
αTatC
100 -
70 -
55 -
40 -
35 -
25 -
4 x TatC
2 x TatC x TatA ?
2 x TatC
TatC x TatA
TatC
1 2 3 4 5 6 7 8 9 10 11 12 13 14 15 16 17 18 19 20
c
INV
TatAC^{Bpa}
129
133
136
157
198
202
205
206
209
UV
αTatA
100 -
70 -
55 -
40 -
35 -
25 -
15 -
TatC x TatA
TatA
1 2 3 4 5 6 7 8 9 10 11 12 13 14 15 16 17 18

Supplementary Figure 5. TatB is required for tetramerization of TatC through residue 133, whereas it impairs interaction between TatC and TatA. (**a**) Membrane vesicles (INV) containing the indicated Bpa variants of TatC were probed for inter-TatC cross-links (blue stars). TatB-TatC adducts (black dots) had been identified as such in Fig. 2a and TatA-TatC adducts (squares) are addressed in **b, c**. ΔTat, vesicles lacking the Tat proteins; TatABC, Bpa-free vesicles. (**b, c**) As in **a**, except that membrane vesicles used were devoid of TatB. UV-dependent adducts to the indicated Bpa variants of TatC were probed by antibodies against TatC (αTatC) and TatA (αTatA) revealing the 37 kDa TatA-TatC complexes (squares). TatA dimers formed independently of Bpa, are indicated by a grey arrow head.

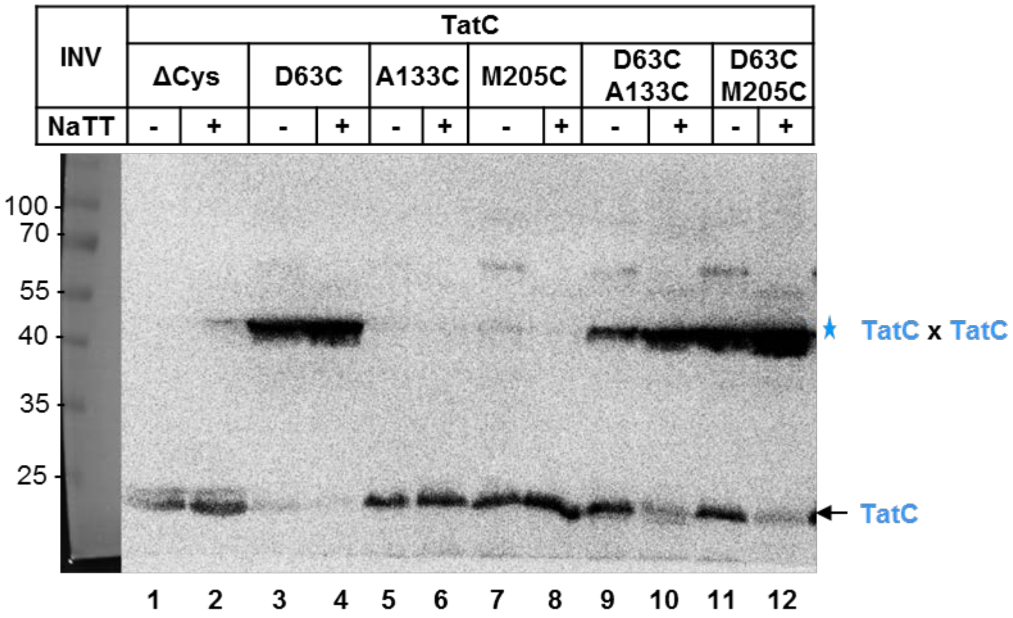

Supplementary Figure 6. No direct cross-linking between the D63 and A133 residues of two adjacent TatC monomers. In contrast to the about 13 Å long, bifunctional cysteine cross-linker Bismaleimidohexane (Fig. 7b), sodium tetrathionate (NaTT) did not yield adducts larger than 70 kDa that would have been indicative of tetramerization and hexamerization of TatC through residues D63 and A133.

Supplementary Figure 7. Uncropped images of Figs. 1-7 and Supplementary Figs. 1-6.

Uncropped Figure 1b

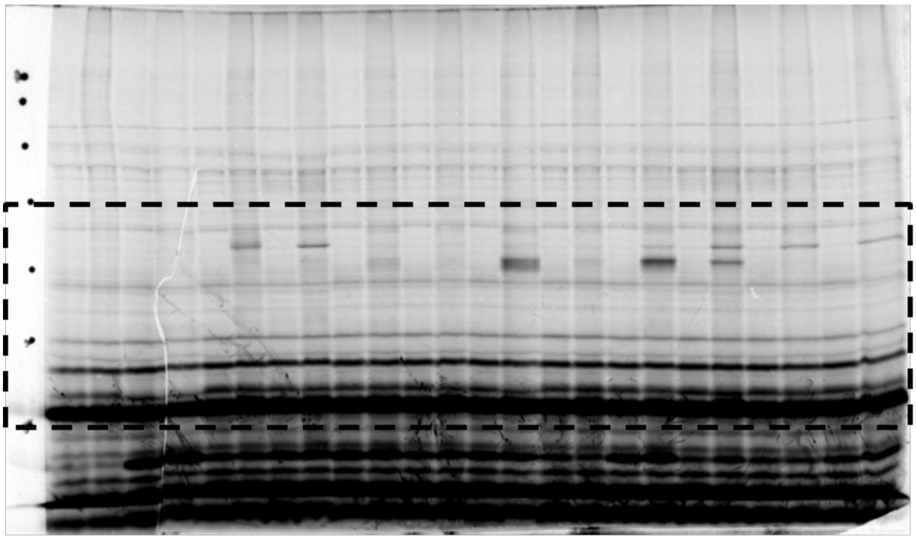

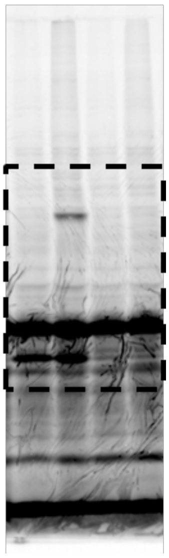

Uncropped Figure 2a

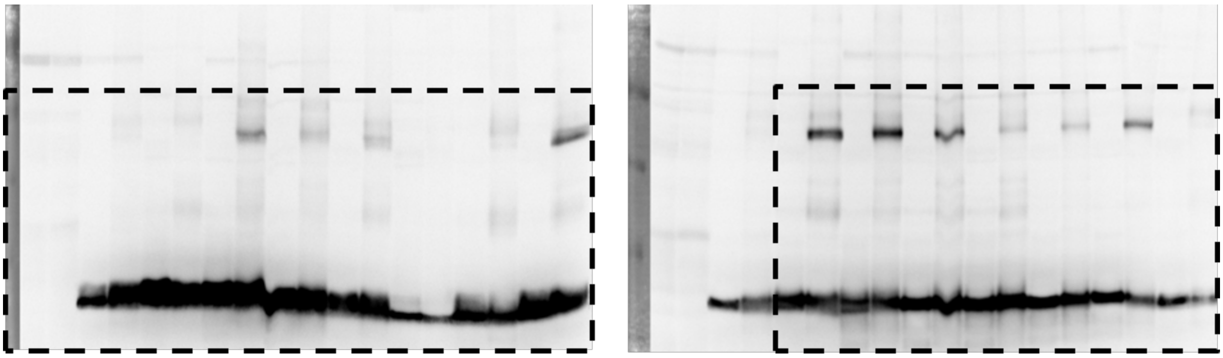

Uncropped Figure 2d

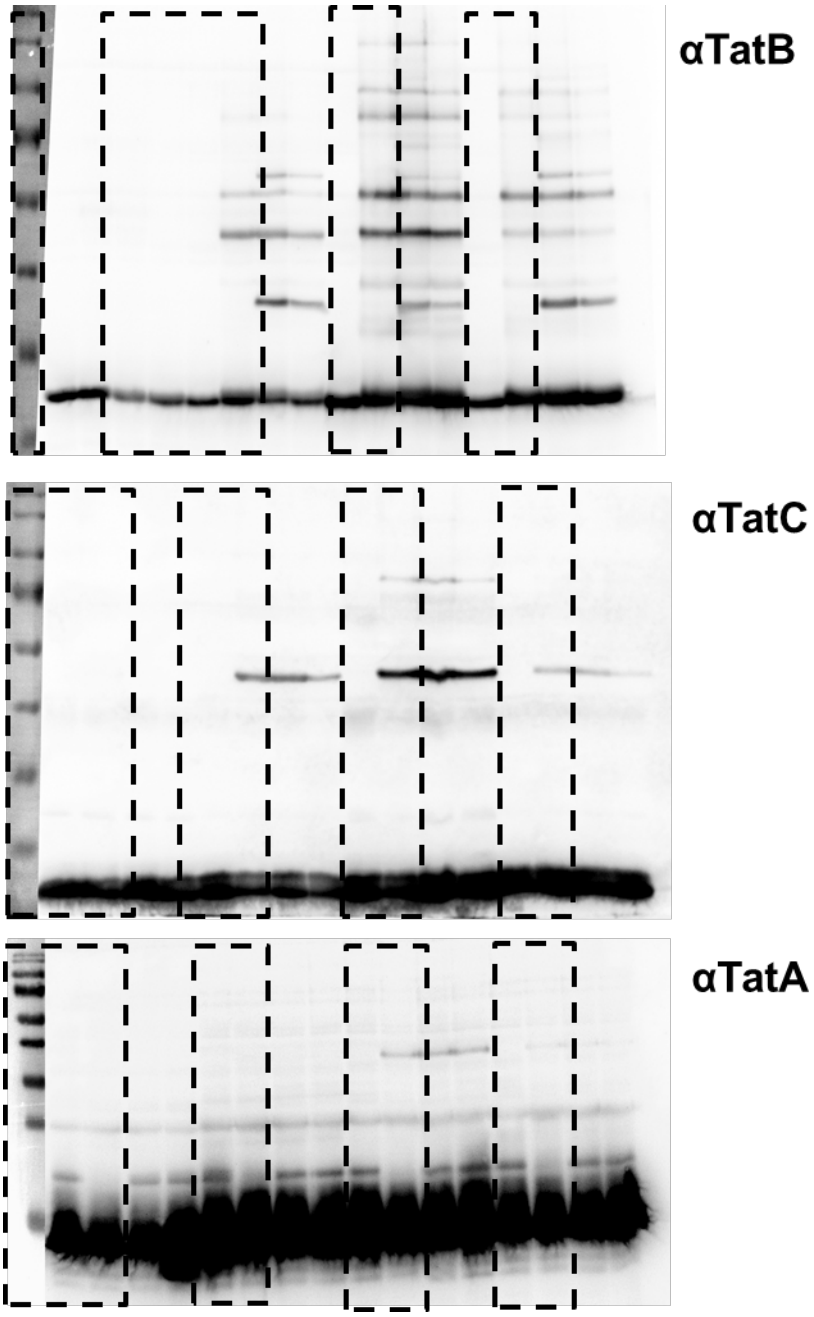

Uncropped Figure 3b

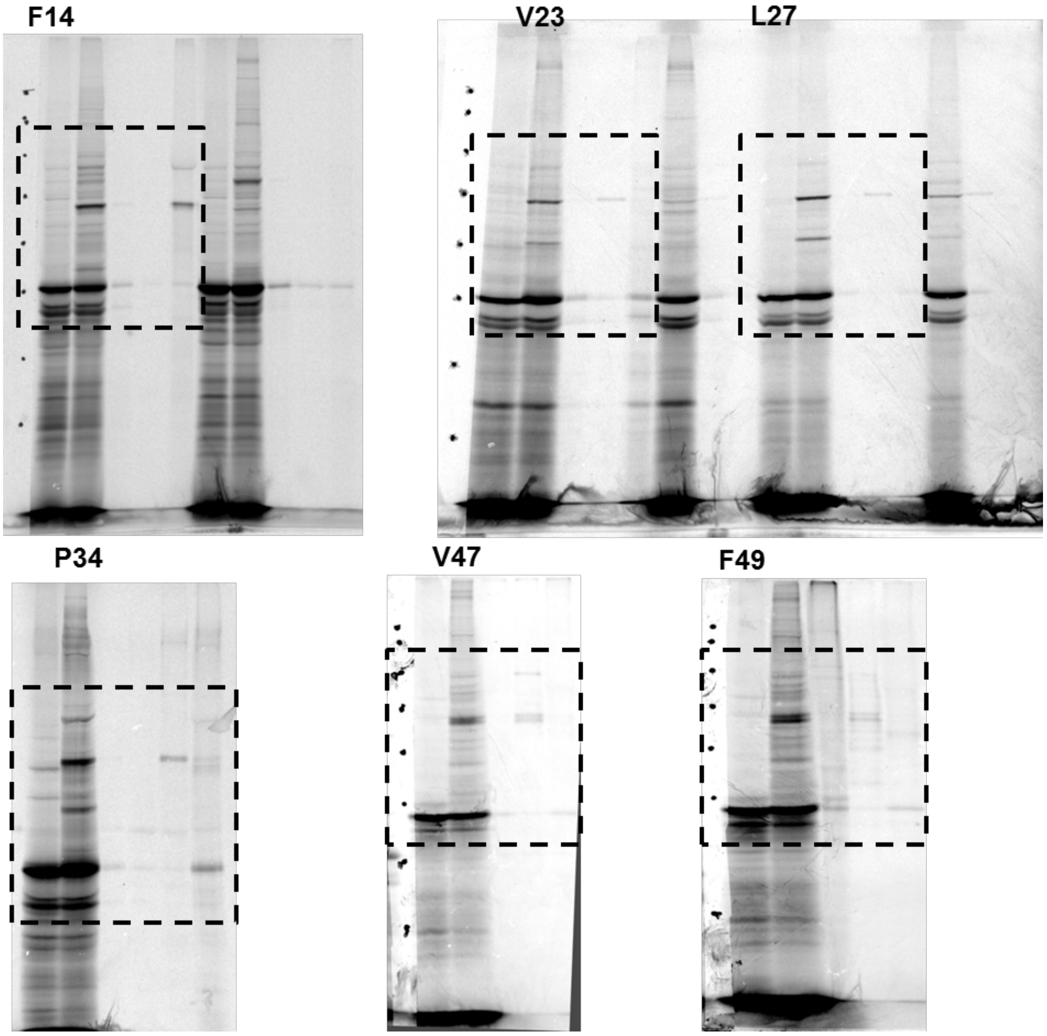

Uncropped Figure 3c

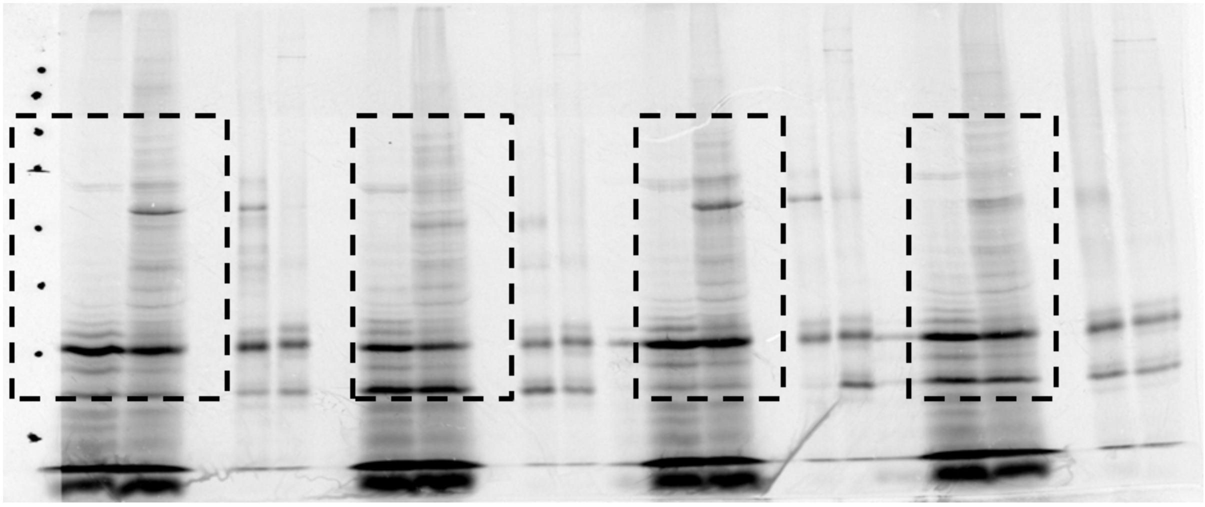

Uncropped Figure 4a

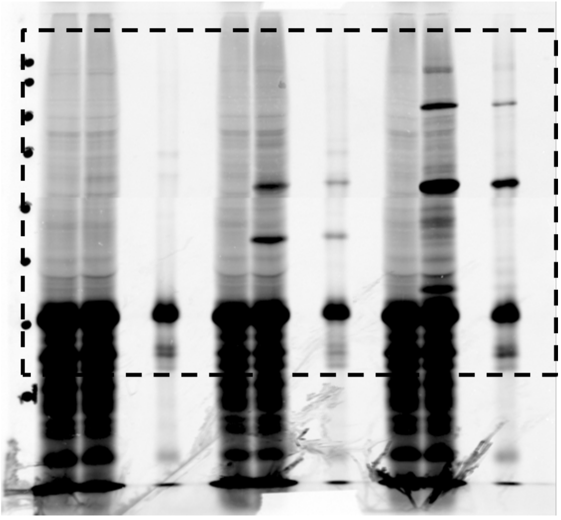

Uncropped Figure 4b

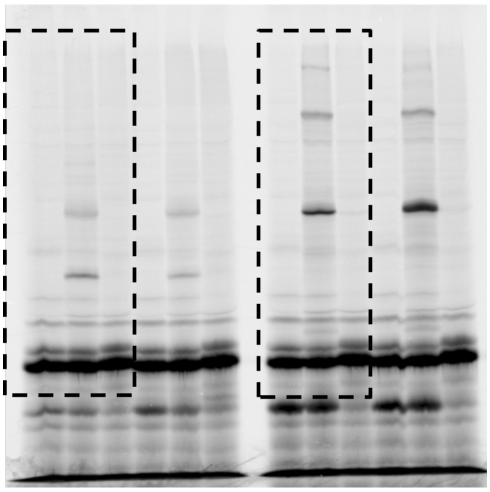

Uncropped Figure 4c

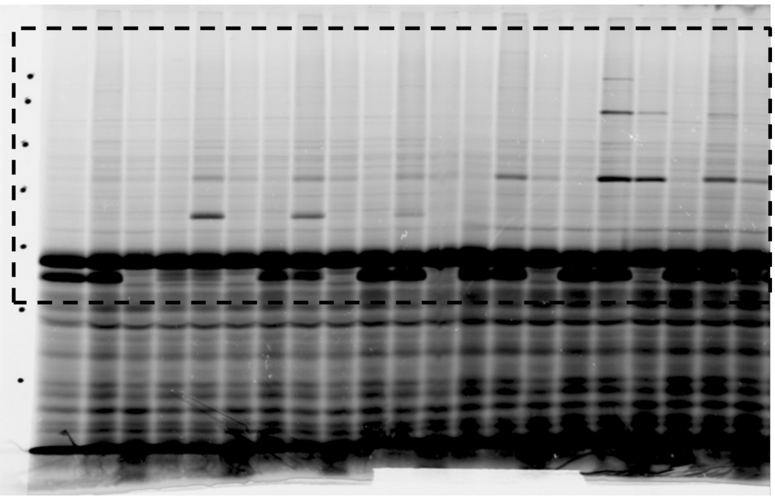

Uncropped Figure 5c

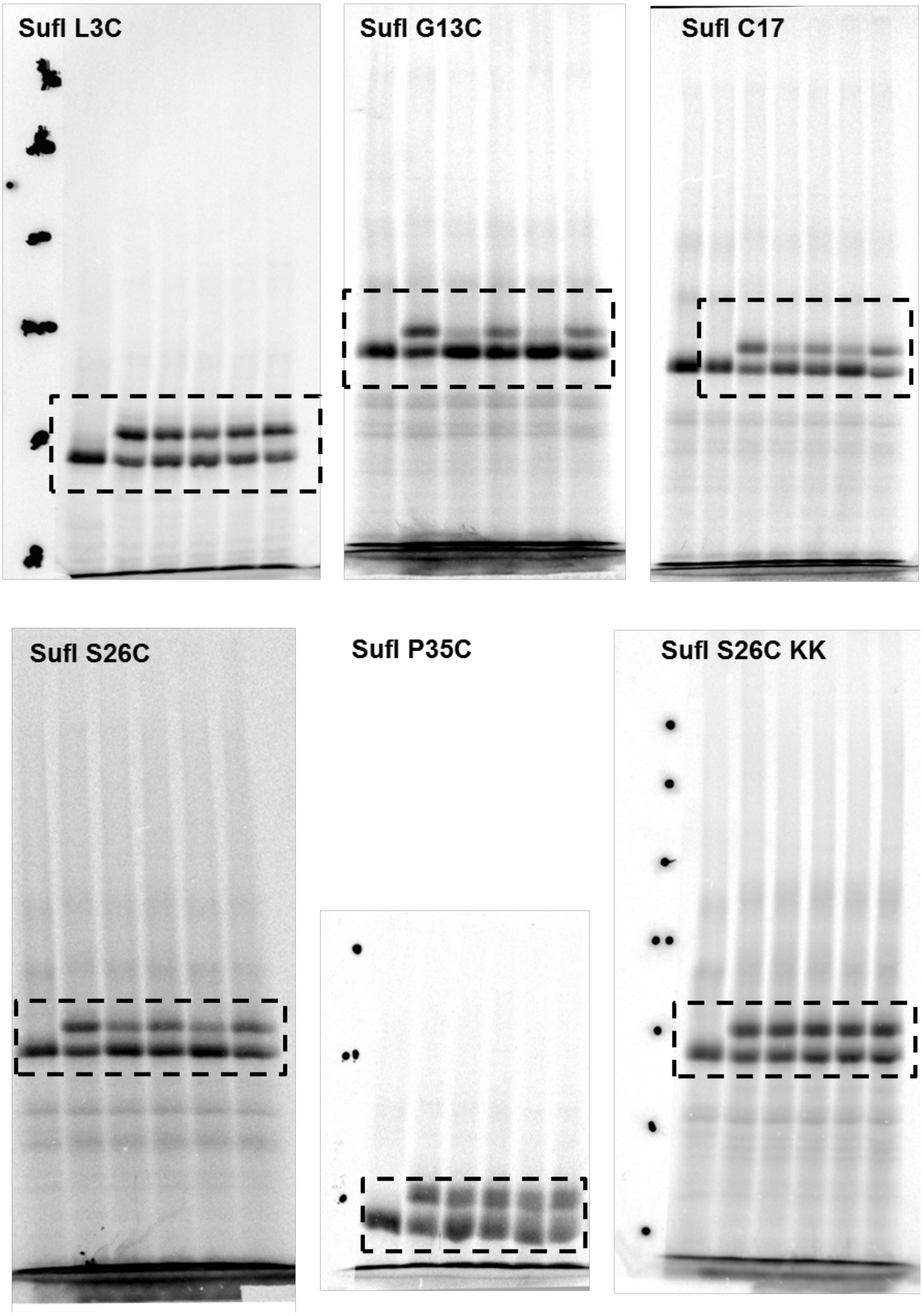

Uncropped Figure 6a

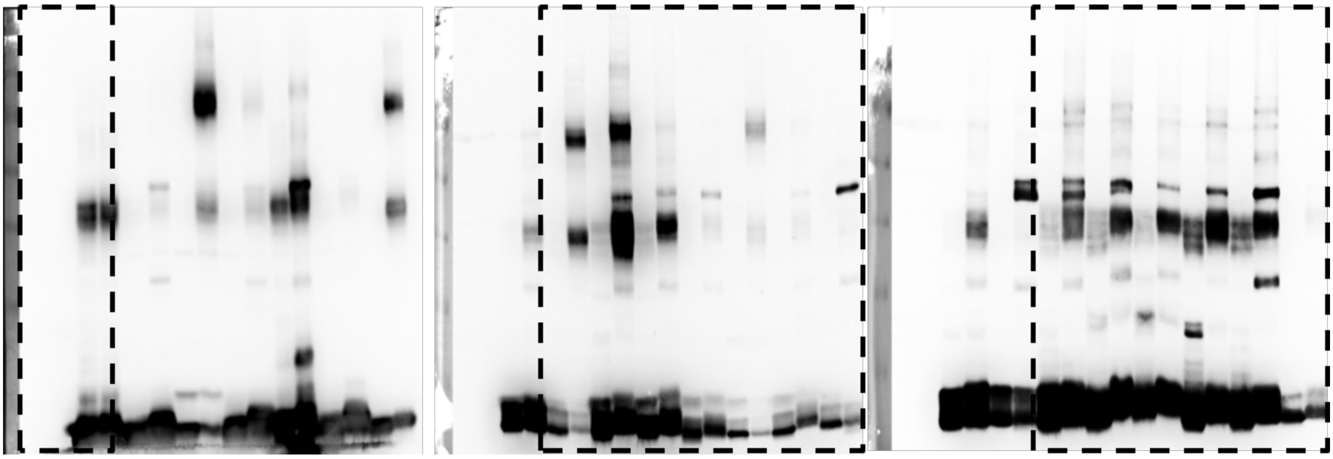

Uncropped Figure 6b

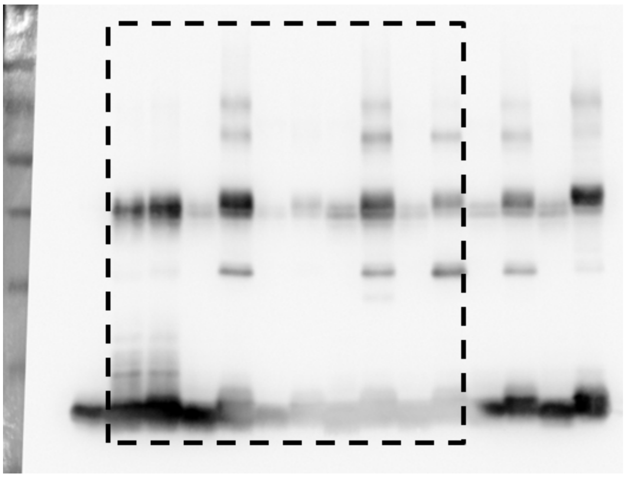

Uncropped Figure 6c

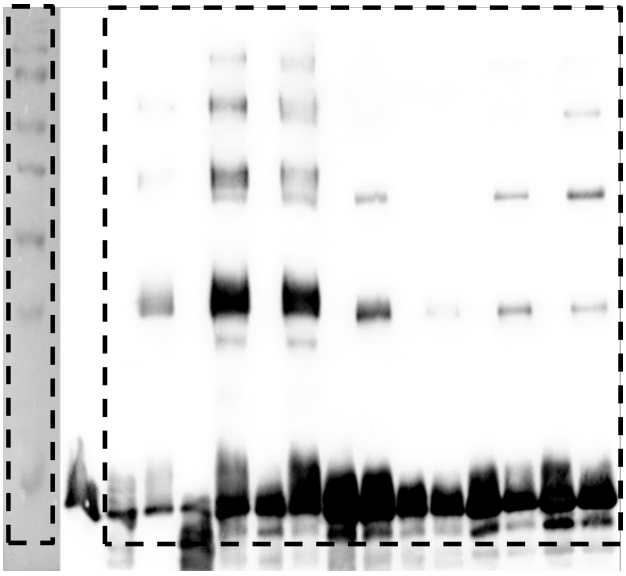

Uncropped Figure 7b

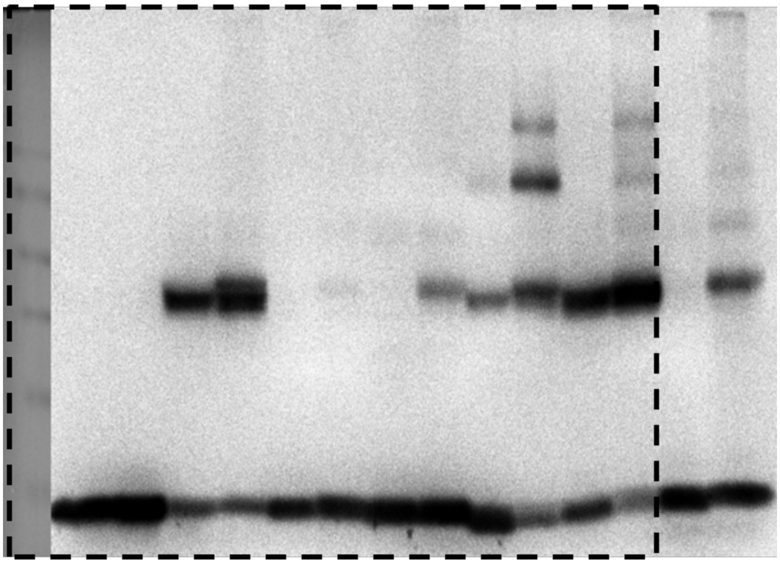

Uncropped Supplementary Figure 1

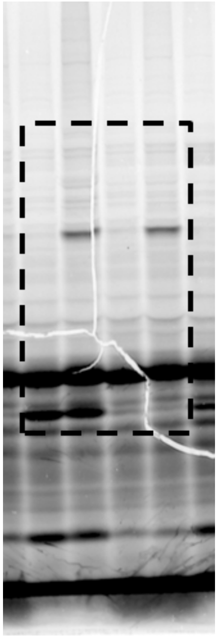

Uncropped Supplementary Figure 2a

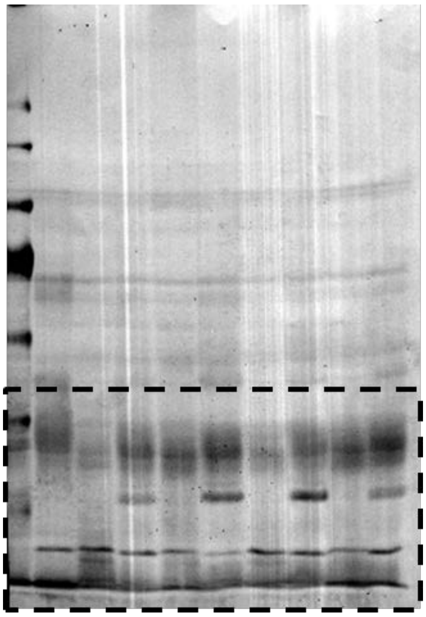

Uncropped Supplementary Figure 2b

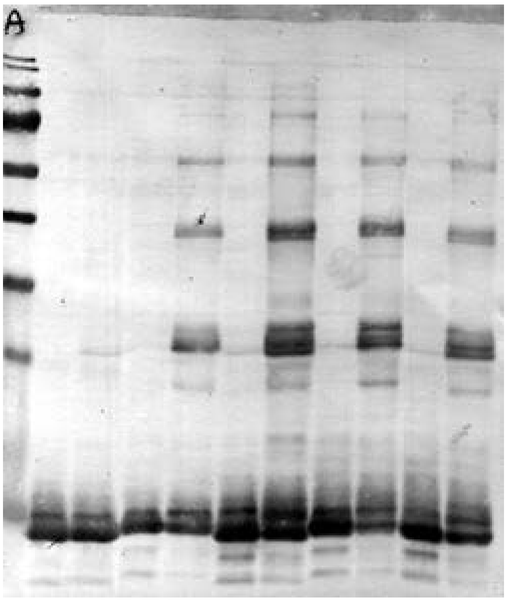

Uncropped Supplementary Figure 3

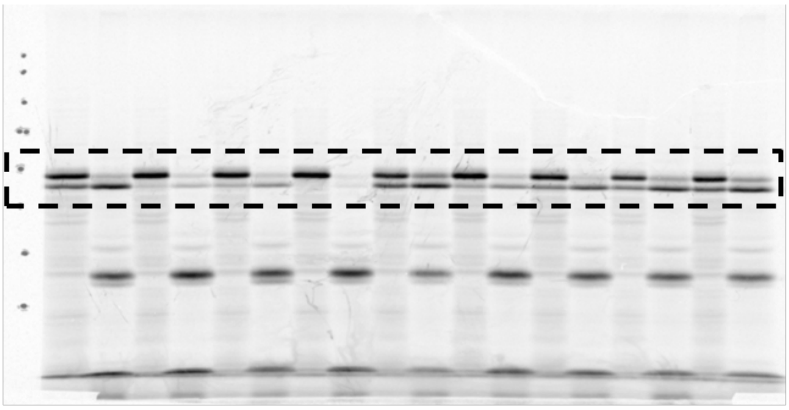

1

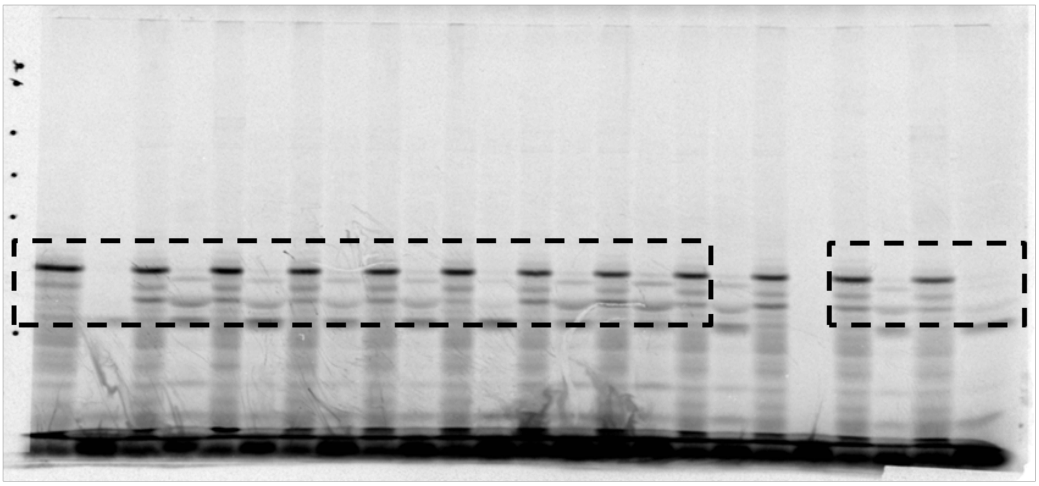

2

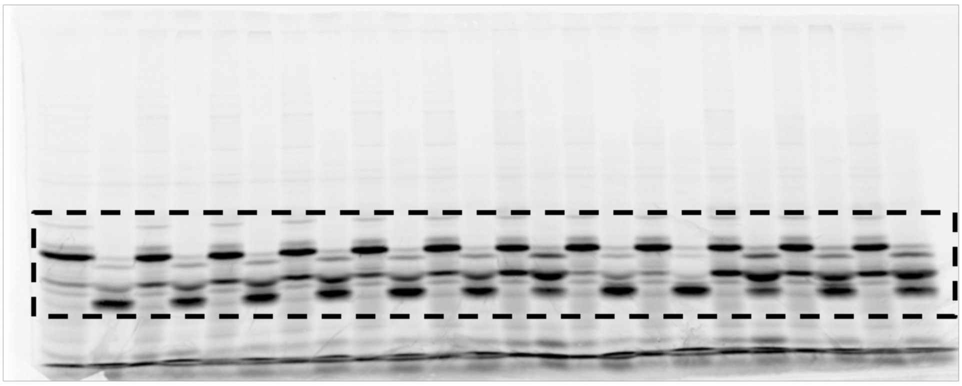

3

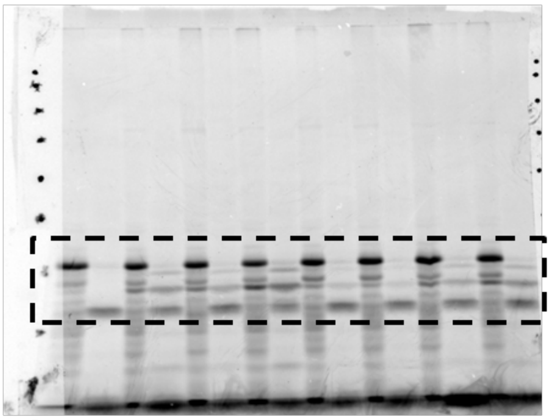

4

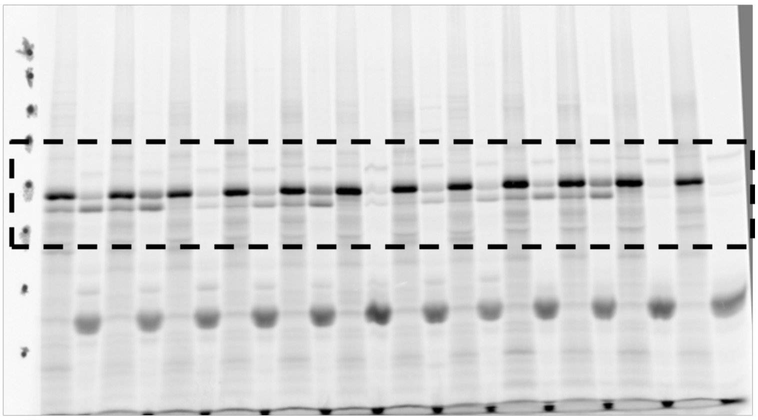

5

Uncropped Supplementary Figure 4

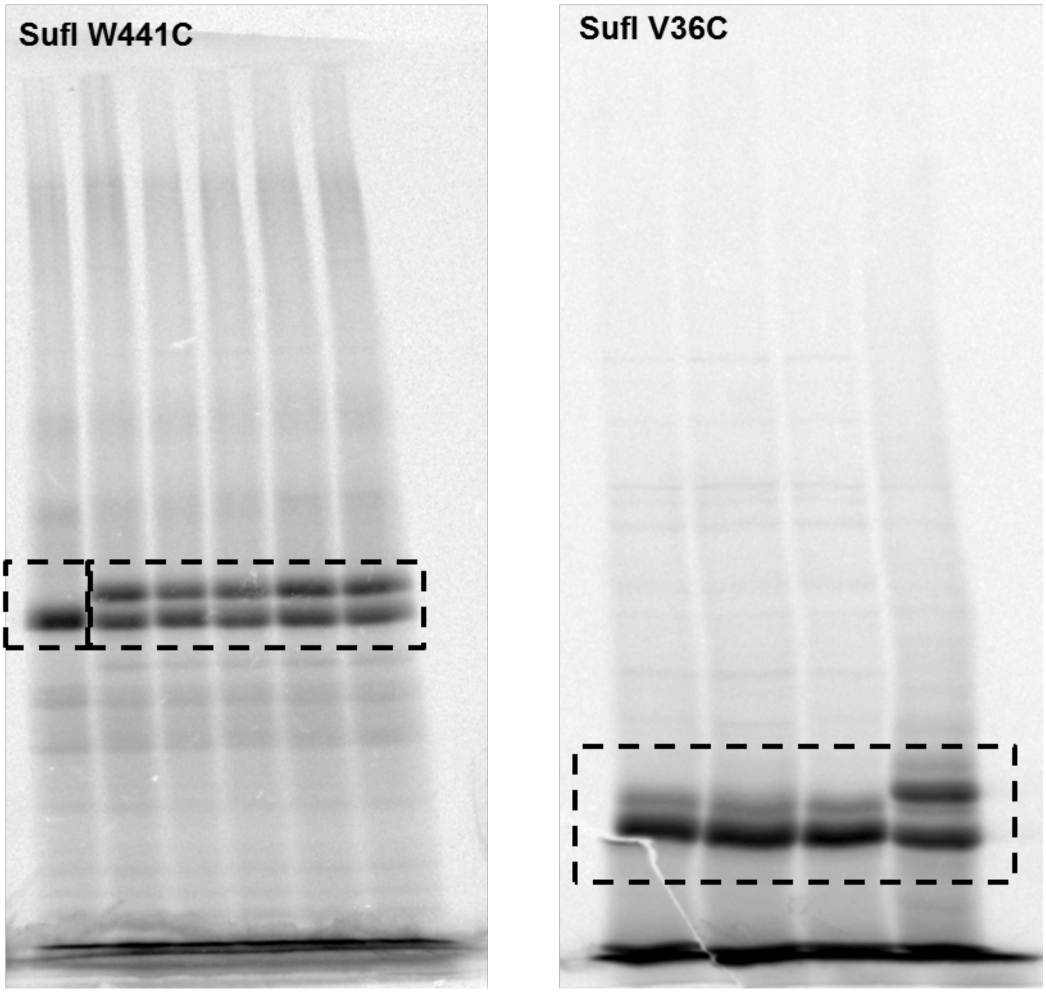

Uncropped Supplementary Figure 5a

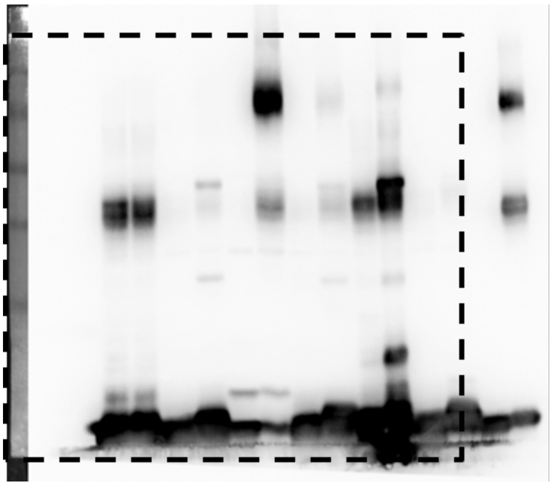

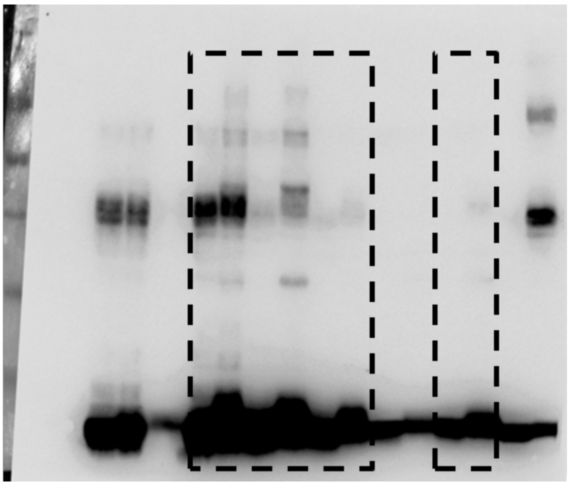

Uncropped Supplementary Figure 5b

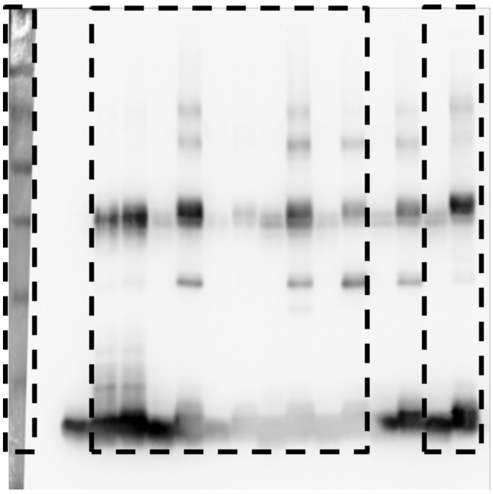

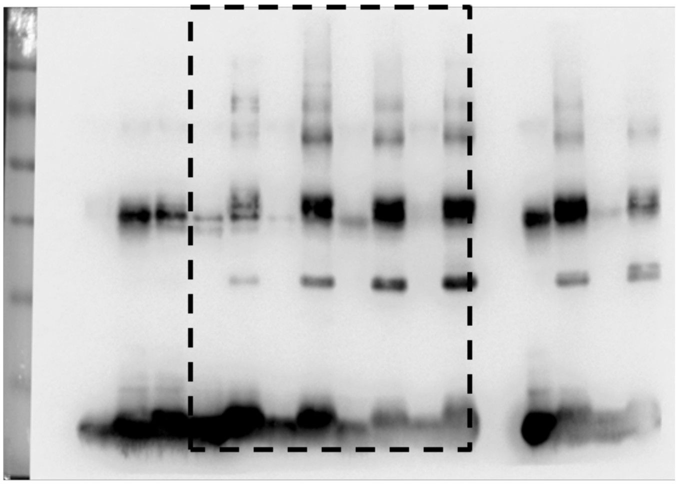

Uncropped Supplementary Figure 5c

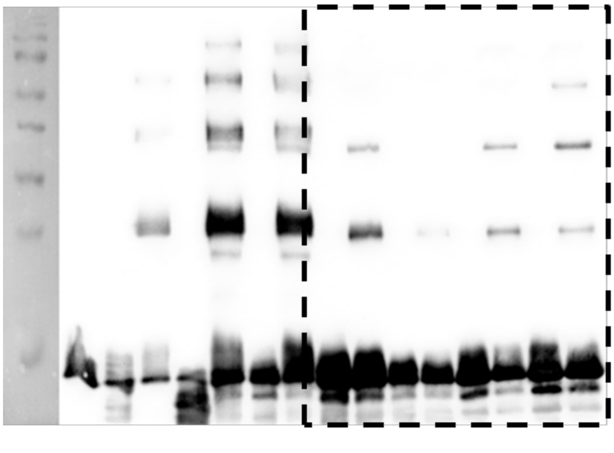

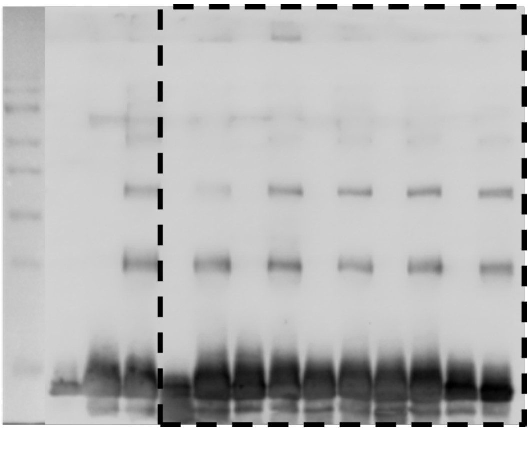

Uncropped Supplementary Figure 6

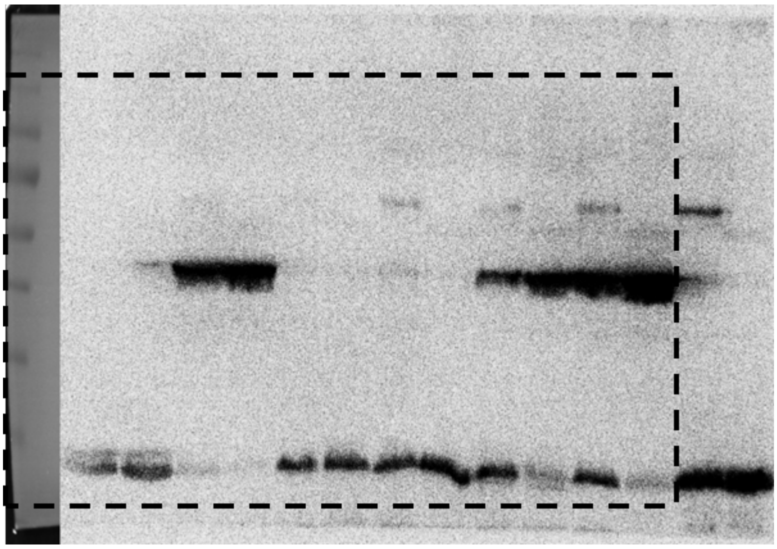

Supplementary Tables

Supplementary Table 1. Quantitative data depicted in Fig. 5b

	Exp.I	Exp.II	Exp.III	mean	S.D.
**ΔTat-INV**					
**L3C**		0.610	0.673	0.642	
**A11C**	0.536	0.607		0.572	
**G13C**	0.468	0.530		0.499	
**II4C**	0.551	0.629		0.590	
**A15C**	0.501	0.656		0.579	
**L16C**	0.417	0.698	0.766	0.627	0.185
**C17**	0.497	0.655	0.648	0.600	0.089
**A18C**	0.617	0.716		0.666	
**G19C**	0.503	0.575		0.539	
**A20C**	0.370	0.532		0.451	
**V21C**	0.556	0.589	0.657	0.600	0.051
**P22C**	0.234	0.258		0.246	
**L23C**	0.538	0.590		0.564	
**A25C**	0.483	0.541		0.512	
**S26C**	0.442	0.481		0.461	
**A28C**	0.502	0.350		0.426	
**G29C**	0.491	0.347		0.419	
**Q30C**	0.293	0.640		0.466	
**Q31C**	0.314	0.155		0.234	
**Q32C**	0.147	0.258		0.202	
**P33C**	0.116	0.094		0.105	
**L34C**	0.148	0.081		0.114	
**P35C**	0.500	0.454	0.720	0.558	0.142
**V36C**	0.062	0.046		0.054	
**W441C**	0.492	0.532		0.512	
**S26CKK**	0.530	0.349		0.439	

Supplementary Table 2. Quantitative data depicted in Fig. 5d

	Exp.I	Exp.II	Exp.III	mean	S.D.
**ΔTat-INV**					
**L3C**		0.610	0.673	0.642	
**G13C**	0.468	0.530		0.499	
**C17**	0.497	0.655	0.648	0.600	0.089
**S26C**	0.442	0.481		0.461	
**P35C**	0.500	0.454	0.720	0.558	
**W441C**	0.492	0.532		0.512	
**S26CKK**	0.530	0.349		0.439	0.128
**TatABC-INV**					
**L3C**		0.476	0.515	0.495	
**G13C**	0.152	0.137		0.145	
**C17**	0.244	0.378	0.298	0.307	0.068
**S26C**	0.218	0.263		0.240	
**P35C**	0.394	0.331	0.500	0.408	
**W441C**	0.418	0.419		0.418	
**S26CKK**	0.525	0.394		0.459	0.093
**TatAC-INV**					
**L3C**		0.413	0.476	0.444	
**G13C**	0.274	0.266		0.270	
**C17**	0.337	0.373	0.270	0.326	0.052
**S26C**	0.339	0.342		0.340	
**P35C**	0.446	0.333	0.328	0.369	
**W441C**	0.459	0.400		0.429	
**S26CKK**	0.504	0.408		0.456	0.067
**TatBC-INV**					
**L3C**		0.497	0.553	0.525	
**G13C**	0.127	0.109		0.118	
**C17**	0.216	0.269	0.278	0.254	0.033
**S26C**	0.132	0.203		0.167	
**P35C**	0.388	0.221	0.414	0.341	
**W441C**	0.545	0.593		0.569	
**S26CKK**	0.492	0.445		0.469	0.033
**TatC-INV**					
**L3C**		0.573	0.618	0.596	
**G13C**	0.302	0.311		0.307	
**C17**	0.446	0.446	0.516	0.469	0.041
**S26C**	0.423	0.394		0.409	
**P35C**	0.500	0.389	0.535	0.474	
**W441C**	0.545	0.514		0.529	
**S26CKK**	0.494	0.383		0.438	0.079

Supplementary Table 3. Plasmids used in this study

Name	Vector	Insert	Reference
pSup-BpaRS-6TRN(D286R)			1
pEVOL-pBpF			2
p8737	pET22b+	TatABCD	3
p8737-tatAC	pET22b+	TatAC	4
pFAT588	pQE	TatCHis	5
pFAT75CHΔA	pQE	TatBCHis	
pPJ3	pET22b+	TorA-mCherry	6
pPJ5	pET22b+	TorA(KK)-mCherry	
pPJ11	pET22b+	TorA-MalE	7
pKSM SufI-RR	pKSM	SufI	8
pEJ	pET22b+	SufI	this study
pETRick	pET22b+	SufI(KK)	
pLJ1	pET22b+	SufIΔCys	
pLJ2	pET22b+	SufI(KK)ΔCys	
pUNITATCC4	pQE60	TatABC ΔCys	9

1. Ryu, Y. & Schultz, P.G. Efficient incorporation of unnatural amino acids into proteins in *Escherichia coli*. *Nat Methods* **3**, 263-265 (2006).
2. Young, T.S., Ahmad, I., Yin, J.A. & Schultz, P.G. An enhanced system for unnatural amino acid mutagenesis in *E. coli*. *J Mol Biol* **395**, 361-374 (2010).
3. Alami, M., Trescher, D., Wu, L.F. & Müller, M. Separate analysis of twin-arginine translocation (Tat)-specific membrane binding and translocation in Escherichia coli. *J Biol Chem* **277**, 20499-20503 (2002).
4. Fröbel, J. et al. Transmembrane insertion of twin-arginine signal peptides is driven by TatC and regulated by TatB. *Nat Commun* **3**, 1311 (2012).
5. Orriss, G.L. et al. TatBC, TatB, and TatC form structurally autonomous units within the twin arginine protein transport system of Escherichia coli. *FEBS Lett* **581**, 4091-4097 (2007).
6. Fröbel, J., Rose, P. & Müller, M. Early contacts between substrate proteins and TatA translocase component in twin-arginine translocation. *J Biol Chem* **286**, 43679-43689 (2011).
7. Zoufaly, S. et al. Mapping Precursor-binding Site on TatC Subunit of Twin Arginine-specific Protein Translocase by Site-specific Photo Cross-linking. *J Biol Chem* **287**, 13430-13441 (2012).
8. Alami, M. et al. Differential interactions between a twin-arginine signal peptide and its translocase in *Escherichia coli*. *Mol Cell* **12**, 937-946 (2003).
9. Lee, P.A. et al. Cysteine-scanning mutagenesis and disulfide mapping studies of the conserved domain of the twin-arginine translocase TatB component. *J Biol Chem* **281**, 34072-85 (2006).

Supplementary Table 4. List of primers (mutations are given in bold)

SufI NdeI	for	GCCATATGTCACTCAGTCGGCG
SufI XhoI	rev	GCCTCGAGTACCGGATTGACC
SufI KK F	for	CATATGTCACTCAGTAAGAAGCAGTTCATTCAGGCATCGG
SufI KK R	rev	CCGATGCCTGAATGAACTGCTTCTTACTGAGTGACATATG
TatA G3	for	GGAACATGTATGGGT**TAG**ATCAGTATTTGGCAG
	rev	CTGCCAAATACTGAT**CTA**ACCCATACATGTTCC
TatA I4	for	CATGTATGGGTGGT**TAG**AGTATTTGGCAGTTATTG
	rev	CAATAACTGCCAAATACT**CTA**ACCACCCATACATG
TatA S5	for	GTATGGGTGGTATC**TAG**ATTTGGCAGTTATTG
	rev	CAATAACTGCCAAAT**CTA**GATACCACCCATAC
TatA I6	for	GGTGGTATCAGT**TAG**TGGCAGTTATTGATTATTG
	rev	CAATAATCAATAACTGCCA**CTA**ACTGATACCACC
TatB F2	for	GCAGGTGTAATCCGTG**TAG**GATATCGGTTTTAGCG
	rev	CGCTAAAACCGATATC**CTA**CACGGATTACACCTGC
TatB I4	for	GGTGTAATCCGTGTTTGAT**TAG**GGTTTTAGCGAACTGC
	rev	GCAGTTCGCTAAAACC**CTA**ATCAAACACGGATTACACC
TatB F6	for	CCGTGTTTGATATCGGT**TAG**AGCGAACTGCTATTGG
	rev	CCAATAGCAGTTCGCT**CTA**ACCGATATCAAACACGG
TatC F69	for	GGCCTCGCCGTTC**TAG**ACGCCGATCAAGC
	rev	GCTTGATCGGCGT**CTA**GAACGGCGAGGCC
TatC F76	for	GCCGATCAAGCTGACC**TAG**ATGGTGTCGCTGATTC
	rev	GAATCAGCGACACCAT**CTA**GGTCAGCTTGATCGGC
TatC L82	for	GGTGTCGCTGATT**TAG**TCAGCGCCGGTG
	rev	CACCGGCGCTGA**CTA**AATCAGCGACACC
TatC I87	for	CTGTCAGCGCCGGTG**TAG**CTCTATCAGGTGTGG
	rev	CCACACCTGATAGAG**CTA**CACCGGCGCTGACAG
TatC V91	for	CCGGTGATTCTCTATCAG**TAG**TGGGCATTTATCGCCCC
	rev	GGGGCGATAAATGCCCA**CTA**CTGATAGAGAATCACCGG
TatC L116	for	GCTGCTGGTTTCCAGCTCT**TAG**CTGTTTTATATCGGCATGG
	rev	CCATGCCGATATAAAACAG**CTA**AGAGCTGGAAACCAGCAGC
TatC V129	for	GCATTCGCCTACTTTGTG**TAG**TTTCCGCTGGCATTTGG
	rev	CCAAATGCCAGCGGAAA**CTA**CACAAAGTAGGCGAATGC
TatC L132	for	CTACTTTGTGGTCTTTCCG**TAG**GCATTTGGCTTCCTTGCC
	rev	GGCAAGGAAGCCAAATGC**CTA**CGGAAAGACCACAAAGTAG
TatC A133	for	CTTTGTGGTCTTTCCGCTG**TAG**TTTGGCTTCCTTGCC
	rev	GGCAAGGAAGCCAAA**CTA**CAGCGGAAAGACCACAAAG
TatC F136	for	CTTTCCGCTGGCATTTGGC**TAG**CTTGCCAATACCGCG
	rev	CGCGGTATTGGCAAG**CTA**GCCAAATGCCAGCGGAAAG
TatC F157	for	CGCCAGCTATTTAAGC**TAG**GTTATGGCGCTGTTTATGG
	rev	CCATAAACAGCGCCATAAC**CTA**GCTTAAATAGCTGGCG
TatC A160	for	CTATTTAAGCTTCGTTATG**TAG**CTGTTTATGGCGTTTGG
	rev	CCAAACGCCATAAACAG**CTA**CATAACGAAGCTTAAATAG
TatC A164	for	CTTCGTTATGGCGCTGTTTATG**TAG**TTTGGTGTCTCCTTTGA AGTGC
	rev	GCACTTCAAAGGAGACACCAAA**CTA**CATAAACAGCGCCATA

		ACGAAG
TatC F165	for	GGCGCTGTTTATGGCG**TAG**GGTGTCTCCTTTGAAGTGC
	rev	GCACTTCAAAGGAGACACC**CTA**CGCCATAAACAGCGCC
TatC S168	for	GTTTATGGCGTTTGGTGTC**TAG**TTTGAAGTGCCGGTAG
	rev	CTACCGGCACTTCAAA**CTA**GACACCAAACGCCATAAAC
TatC L177	for	GAAGTGCCGGTAGCAATTGTG**TAG**CTGTGCTGGATGGG
	rev	CCCATCCAGCACAG**CTA**CACAATTGCTACCGGCACTTC
TatC W180	for	GCAATTGTGCTGCTGTGC**TAG**ATGGGGATTACCTCGCC
	rev	GGCGAGGTAATCCCCAT**CTA**GCACAGCAGCACAATTGC
TatC M181	for	GCAATTGTGCTGCTGTGCTGG**TAG**GGGATTACCTCGCC
	rev	GGCGAGGTAATCCC**CTA**CCAGCACAGCAGCACAATTGC
TatC V198	for	CAAAAAACGCCCGTATGTGCTG**TAG**GGTGCATTCGTTG
	rev	CAACGAATGCACC**CTA**CAGCACATACGGGCGTTTTTTG
TatC V202	for	GTGCTGGTTGGTGCATTC**TAG**GTCGGGATGTTGCTGAC
	rev	GTCAGCAACATCCCGAC**CTA**GAATGCACCAACCAGCAC
TatC M205	for	GTTGGTGCATTCGTTGTCGGG**TAG**TTGCTGACGCCGCC
	rev	GGCGGCGTCAGCAA**CTA**CCCGACAACGAATGCACCAAC
TatC L206	for	GCATTCGTTGTCGGGATG**TAG**CTGACGCCGCCGGATG
	rev	CATCCGGCGGCGTCAG**CTA**CATCCCGACAACGAATGC
TatC T208	for	GTTGTCGGGATGTTGCTG**TAG**CCGCCGGATGTCTTCTC
	rev	GAGAAGACATCCGGCGG**CTA**CAGCAACATCCCGACAAC
TatC P209	for	GTTGTCGGGATGTTGCTGACG**TAG**CCGGATGTCTTCTC
	rev	GAGAAGACATCCGG**CTA**CGTCAGCAACATCCCGACAAC
TatC F213	for	GCTGACGCCGCCGGATGTC**TAG**TCGCAAACGCTGTTGG
	rev	CCAACAGCGTTTGCGA**CTA**GACATCCGGCGGCGTCAGC
TatC T216	for	GGATGTCTTCTCGCAA**TAG**CTGTTGGCGATCCCG
	rev	CGGGATCGCCAACAG**CTA**TTGCGAGAAGACATCC
TatC I220	for	CGCAAACGCTGTTGGCG**TAG**CCGATGTACTGTCTGTTTG
	rev	CAAACAGACAGTACATCGG**CTA**CGCCAACAGCGTTTGCG
TatC Y223	for	GCTGTTGGCGATCCCGATG**TAG**TGTCTGTTTGAAATCGGTG
	rev	CACCGATTTCAAACAGACA**CTA**CATCGGGATCGCCAACAGC
TatC A133C	for	CTACTTTGTGGTCTTTCCGCTG**TGT**TTTGGCTTCCTTGC
	rev	GCAAGGAAGCCAAA**ACA**CAGCGGAAAGACCACAAAGTAG
TatC D63C	for	GTTCAACGATGATCGCCACC**TGT**GTGGCCTCGCCGTTC
	rev	GAACGGCGAGGCCAC**ACA**GGTGGCGATCATCGTTGAAC
TatC M205C	for	GGTGCATTCGTTGTCGGG**TGT**TTGCTGACGCCGCCG
	rev	CGGCGGCGTCAGCAA**ACA**CCCGACAACGAATGCACC
TorA SS F14_f	for	CATCACGTCGGCGT**TAG**CTGGCACAACTCGGCGGC
TorA SS F14_r	rev	GCCGCCGAGTTGTGCCAG**CTA**ACGCCGACGTGATG
TorA SS V23_f	for	CAACTCGGCGGCTTAACC**TAG**GCCGGGATGCTGG
TorA SS V23_r	rev	CCAGCATCCCGGC**CTA**GGTTTAGCCGCCGAGTTG
TorA SS L27_f	for	CCGTCGCCGGGATG**TAG**GGGCCGTCATTGTTAACGC

TorA SS L27_r	rev	GCGTTAACAATGACGGCCC**CTA**CATCCCGGCGACGG
TorA SS P34_f	for	CCGTCATTGTTAACG**TAG**CGACGTGCGACTGCG
TorA SS P34_r	rev	CGCAGTCGCACGTCG**CTA**CGTTAACAATGACGG
TorA-MalE V47	for	GCGGCGACTGACGCT**TAG**GAATTCGATATCATCGAAG
	rev	CTTCGATGATATCGAATTC**CTA**AGCGTCAGTCGCCGC
TorA-MalE F49	for	CGACTGACGCTGTCGAA**TAG**GATATCATCGAAGAAGG
	rev	CCTTCTTCGATGATATC**CTA**TTCGACAGCGTCAGTCG
TorA-mCherry V47	for	GCGGCGACTGACGCT**TAG**GAATTCATGGTGAGCAAGGG
	rev	CCCTTGCTCACCATGAATTC**CTA**AGCGTCAGTCGCCGC
TorA-mCherry F49	for	GCGACTGACGCTGTCGAA**TAG**ATGGTGAGCAAGGGC
	rev	GCCCTTGCTCACCAT**CTA**TTCGACAGCGTCAGTCGC
SufI C17A	for	GGG ATT GCA CTT **GCG** GCA GGC GCT G
	rev	C AGC GCC TGC **CGC** AAG TGC AAT CCC
SufI C295A	for	GTG TCG ATC ACC **GCG** GGC GAA GCG G
	rev	C CGC TTC GCC **CGC** GGT GAT CGA CAC
SufI L3C	for	GGAGATATACATATGTCA**TGC**AGTCGGCGTCAGTTCATTC
	rev	GAATGAACTGACGCCGACT**GCA**TGACATATGTATATCTCC
SufI A11C	for	CGTCAGTTCATTCAG**TGC**TCGGGGATTGCACTTGCG
	rev	CGCAAGTGCAATCCCCGA**GCA**CTGAATGAACTGACG
SufI G13C	for	GTCAGTTCATTCAGGCATCG**TGC**ATTGCACTTGCGGCAGG
	rev	CCTGCCGCAAGTGCAAT**GCA**CGATGCCTGAATGAACTGAC
SufI I14C	for	CAGGCATCGGGG**TGT**GCACTTGCGGCAGGC
	rev	GCCTGCCGCAAGTGC**ACA**CCCCGATGCCTG
SufI A15C	for	GGCATCGGGGATT**TGC**CTTGCGGCAGGCG
	rev	CGCCTGCCGCAAG**GCA**AATCCCCGATGCC
SufI L16C	for	CGGGGATTGCA**TGT**GCGGCAGGCGC
	rev	GCGCCTGCCGC**ACA**TGCAATCCCCG
SufI A18C	for	GGGATTGCACTTGCG**TGC**GGCGCTGTTCCCC
	rev	GGGGAACAGCGCC**GCA**CGCAAGTGCAATCCC
SufI G19C	for	GGATTGCACTTGCGGCA**TGC**GCTGTTCCCCTGAAGG
	rev	CCTTCAGGGGAACAGC**GCA**TGCCGCAAGTGCAATCC
SufI A20C	for	GGGATTGCACTTGCGGCAGGC**TGT**GTTCCCCTGAAGGCC
	rev	GGCCTTCAGGGGAAC**ACA**GCCTGCCGCAAGTGCAATCCC
SufI V21C	for	GCACTTGCGGCAGGCGCT**TGT**CCCCTGAAGGCCAGC
	rev	GCTGGCCTTCAGGGG**ACA**AGCGCCTGCCGCAAGTGC
SufI P22C	for	GCGGCAGGCGCTGTT**TGC**CTGAAGGCCAGCGC
	rev	GCGCTGGCCTTCAG**GCA**AACAGCGCCTGCCGC
SufI L23C	for	GCAGGCGCTGTTCCC**TGC**AAGGCCAGCGCAGC
	rev	GCTGCGCTGGCCTT**GCA**GGGAACAGCGCCTGC
SufI A25C	for	GCTGTTCCCCTGAAG**TGC**AGCGCAGCCGGG
	rev	CCCGGCTGCGCT**GCA**CTTCAGGGGAACAGC
SufI S26C	for	CCTGAAGGCC**TGT**GCAGCCGGGCAAC
	rev	GTTGCCCGGCTGC**ACA**GGCCTTCAGG
SufI A28C	for	CCTGAAGGCCAGCGCA**TGC**GGGCAACAGCAACC

	rev	GGTTGCTGTTGCCC**GCA**TGCGCTGGCCTTCAGG
SufI G29C	for	GGCCAGCGCAGCC**TGC**CAACAGCAACCGC
	rev	GCGGTTGCTGTTG**GCA**GGCTGCGCTGGCC
SufI Q30C	for	GCGCAGCCGGG**TGC**CAGCAACCGCTACC
	rev	GGTAGCGGTTGCTG**GCA**CCCGGCTGCGC
SufI Q31C	for	CGCAGCCGGGCAA**TGC**CAACCGCTACCCG
	rev	CGGGTAGCGGTTG**GCA**TTGCCCGGCTGCG
SufI Q32C	for	GCCGGGCAACAG**TGC**CCGCTACCCGTTCCG
	rev	CGGAACGGGTAGCGG**GCA**CTGTTGCCCGGC
SufI P33C	for	GGGCAACAGCAA**TGC**CTACCCGTTCCGCCG
	rev	CGGCGGAACGGGTAG**GCA**TTGCTGTTGCCC
SufI L34C	for	GGCAACAGCAACCG**TGC**CCCGTTCCGCCGC
	rev	GCGGCGGAACGGG**GCA**CGGTTGCTGTTGCC
SufI P35C	for	GCAACAGCAACCGCTA**TGC**GTTCCGCCGCTACTTGAATCTC
	rev	GAGATTCAAGTAGCGGCGGAAC**GCA**TAGCGGTTGCTGTTGCC
SufI V36C	for	CAACCGCTACCC**TGT**CCGCCGCTACCTG
	rev	CAAGTAGCGGCGG**ACA**GGGTAGCGGTTG
SufI W441C	for	GGTCAGCCTTCC**TGC**GCGCACTTCCCG
	rev	CGGGAAGTGCGC**GCA**GGAAGGCTGACC
